# Characterization of Novel Broad-Host-Range Bacteriophage DLP3 Specific to *Stenotrophomonas maltophilia* as a Potential Therapeutic Agent

**DOI:** 10.3389/fmicb.2020.01358

**Published:** 2020-06-24

**Authors:** Danielle L. Peters, Jaclyn G. McCutcheon, Jonathan J. Dennis

**Affiliations:** Department of Biological Sciences, Faculty of Science, University of Alberta, Edmonton, AB, Canada

**Keywords:** *Stenotrophomonas*, bacteriophage, phage, phage therapy, antimicrobial resistance

## Abstract

A novel *Siphoviridae* phage specific to the bacterial species *Stenotrophomonas maltophilia* was isolated from a pristine soil sample and characterized as a second member of the newly established *Delepquintavirus* genus. Phage DLP3 possesses one of the broadest host ranges of any *S. maltophilia* phage yet characterized, infecting 22 of 29 *S. maltophilia* strains. DLP3 has a genome size of 96,852 bp and a G+C content of 58.4%, which is significantly lower than *S. maltophilia* host strain D1571 (G+C content of 66.9%). The DLP3 genome encodes 153 coding domain sequences covering 95% of the genome, including five tRNA genes with different specificities. The DLP3 lysogen exhibits a growth rate increase during the exponential phase of growth as compared to the wild type strain. DLP3 also encodes a functional erythromycin resistance protein, causing lysogenic conversion of the host D1571 strain. Although a temperate phage, DLP3 demonstrates excellent therapeutic potential because it exhibits a broad host range, infects host cells through the *S. maltophilia* type IV pilus, and exhibits lytic activity *in vivo*. Undesirable traits, such as its temperate lifecycle, can be eliminated using genetic techniques to produce a modified phage useful in the treatment of *S. maltophilia* bacterial infections.

## Introduction

*Stenotrophomonas maltophilia* is a robust, non-sporulating, obligate aerobe Gram-negative bacillus (Brooke, 2012). The genus name *Stenotrophomonas* was originally chosen due to the perceived limited nutritional range of the bacterium (Palleroni and Bradbury, 1993), though many studies have since shown an impressive metabolic versatility (Chatelut et al., 1995; Berg et al., 1999; Hauben et al., 1999; Minkwitz and Berg, 2001; Svensson-Stadler et al., 2012). *S. maltophilia* is often found in close association with plant rhizospheres where they actively promote plant growth through the secretion of growth-promoting compounds (Ryan et al., 2009). This plant growth promotion is now being used commercially; *S. maltophilia* strains are used as biofertilizers due to their ability to fix nitrogen, produce growth-promoting plant hormones, and protect plant roots from phytopathogens (Rathi and Nandabalan, 2017). Additionally, the vast metabolic diversity observed in *S. maltophilia* enables these bacteria to be used in bioremediation, from heavy metal detoxification of soils and waterways to the degradation of insecticides and volatile organic compounds such as benzene. However, their widespread use in biotechnology and agriculture is problematic due to their ability to cause disease in humans (Berg and Martinez, 2015).

*S. maltophilia* is an important multidrug resistant, opportunistic pathogen that is most commonly associated with pneumonia and bacteremia in immunocompromised patients. *S. maltophilia* has also been identified as the cause of soft tissue infections, osteomyelitis, meningitis, endocarditis, otitis and scleritis (Brooke, 2012). Several risk factors are associated with *S. maltophilia* infections in the general population, including malignancy, human immunodeficiency virus, cystic fibrosis (CF), intravenous drug use, surgical and accidental trauma, prolonged hospitalization, mechanical ventilation, indwelling catheters, corticosteroids, immunosuppressive therapy, and treatment with broad-spectrum antibiotics (Al-Anazi and Al-Jasser, 2014). One study estimated the nosocomial mortality rates attributed to *S. maltophilia* bacteremia at 16.7%, and the overall mortality rate of patients infected with *S. maltophilia* was 25% (Naidu and Smith, 2012). A recent study on the clinical outcomes of cancer patients with bloodstream infections (BSI) and pneumonia caused by *S. maltophilia* infections in Mexico City indicated that 31.6% died within the first month; 22.1% of the deaths were due to pneumonia and 9.5% were due to BSI (Velazquez-Acosta et al., 2018). A similar study on BSI mortality rates associated with 937 German intensive care units found *S. maltophilia* infections had the highest mortality rates (28.4%), followed next by non-*albicans*-*Candida spp.* (27.1%) and *Pseudomonas aeruginosa* (25.8%) (Schwab et al., 2018).

Due to a wide array of pathogenicity factors, *S. maltophilia* can be difficult to clear once an infection has been established. *S. maltophilia* pathogenicity mechanisms include swimming and twitching motility (from flagella and type IV pili, respectively), a DNA hypermutator mechanism, iron uptake transporters, biofilm formation, lipopolysaccharide (LPS), many extracellular enzymes encoded such as fibrolysin, lipases, esterase, DNase, RNase, proteases and lecithinase, type II protein secretion systems, host cell invasion, and quorum sensing signaling systems (Brooke, 2012). In addition, clinical *S. maltophilia* isolate culture supernatants applied to Hep-2, HeLa, or Vero cells resulted in cytotoxic effects such as intensive rounding, loss of intercellular junctions, and membrane blebbing followed by cell death within 24 h (Figueiredo et al., 2006). In some cases, endocytosis, hemolytic activity, and cell aggregation were observed, and the addition of protease inhibitors did not prevent cell cytotoxicity. LPS production was shown to be required for attachment to surfaces, and colonization and virulence in a rat lung model (McKay et al., 2003). As well, it was shown that an *O*-polysaccharide synthesis mutant was susceptible to complement-mediated cell killing (Brooke et al., 2008). *S. maltophilia* can form biofilms on many surfaces such as glass, plastics, implanted medical devices, and lung epithelial cells (Huang et al., 2006; Pompilio et al., 2008, 2010; Brooke, 2014). A study focusing on the biofilm formation of CF-derived bronchial epithelial cell monolayers revealed that all *S. maltophilia* isolates were able to form biofilms on bronchial epithelial cells (Pompilio et al., 2010). Type-1 fimbriae genes have only been identified in clinical *S. maltophilia* isolates, which suggests they may play a direct role in the colonization of infected CF individuals (Nicoletti et al., 2011). *In vitro* tissue culture assays have indicated the *S. maltophilia* fimbriae 1 (SMF-1) protein is important for adherence to eukaryotic cells and glass, and anti-SMF-1 antibodies inhibit adherence to eukaryotic cells and glass if the antibodies were applied during early stages of infection (de Oliveira-Garcia et al., 2003). The ability of *S. maltophilia* isolates to form biofilms and invade host cells contributes to the prolonged infections noted in hematology and oncology patients, despite aggressive antibiotic treatments (Lai et al., 2006).

Innate antibiotic resistance in *S. maltophilia* infections is a major contributing factor to treatment failure. Patients who receive the wrong antibiotic at initial infection diagnosis have an increased risk of mortality compared to patients who received appropriate *S. maltophilia* antibiotic treatment initially (Velazquez-Acosta et al., 2018). *S. maltophilia* infections can be further complicated by the emergence of mutants with pleiotropic antibiotic resistance (Denton and Kerr, 1998). Intrinsic resistance is attributed to mechanisms such as reduced membrane permeability (Mett et al., 1988), chromosomally encoded multidrug efflux pumps, and chromosomally encoded Qnr pentapeptide repeat proteins (Sanchez et al., 2008; Sanchez and Martinez, 2010; Chang et al., 2011). Additionally, antibiotic inactivating enzymes such as inducible L1 and L2 β-lactamases (Walsh et al., 1997; Crowder et al., 1998; Okazaki and Avison, 2008), a TEM-2 β-lactamase from a Tn*1*-like transposon (Avison et al., 2000), and aminoglycoside- inactivating enzymes (Okazaki and Avison, 2007; Vetting et al., 2008) have been identified in many *S. maltophilia* isolates. The L1 protein is a molecular class B zinc-dependent metallo-β-lactamase which hydrolyzes virtually all classes of β-lactams, including penicillins, cephalosporins and carbapenems (Avison et al., 2001), whereas the L2 protein is a molecular class A clavulanic acid-sensitive cephalosporinase (Chang et al., 2015). The drug of choice to treat *S. maltophilia* infections continues to be trimethoprim/sulfamethoxazole (TMP/SMX) (Chung et al., 2013; Wang et al., 2016), although resistance to TMP/SMX across clinical isolates is common (30.5%) (Ochoa-Sanchez and Vinuesa, 2017). However, higher rates of TMP/SMX resistance, ranging from 16% to nearly 80%, have been noted in patients with cancer, CF, and in several specific countries (Chang et al., 2015; Rhee et al., 2016). The majority of TMP/SMX resistance is due to the presence of a *sul* gene encoding a sulfonamide resistance protein located on a class 1 integron (sul1) (Barbolla et al., 2004; Chung et al., 2015), and also on insertion sequence common region (ISCR) elements (sul2) (Toleman et al., 2007) rather than through the overexpression of efflux pumps. A *dhfr* gene encoding a dihydrofolate reductase associated with a class 1 integron has also been shown to cause some level of resistance against TMP/SMX (Hu et al., 2011), along with the activity from several RND family efflux pumps, although not to the same degree as the resident *sul* genes (Huang et al., 2013; Lin et al., 2015; Sanchez, 2015). Thus, research into alternative treatment options is critical due to increased resistance to TMP/SMX noted with specific comorbidities and geographic locations.

Related to this high-level antibiotic resistance, *S. maltophilia* is also capable of tolerating biocides and harsh environments, which makes this bacterium difficult to eradicate from hospital settings. Nosocomial isolation sources for *S. maltophilia* include ultra-pure water, hemodialysis water, nebulizers, hand-washing soap, hospital antiseptic solution, chlorhexidine-cetrimide topical antiseptic solution, hypochlorite cleaners, triclosan, sodium dodecyl sulfate, and antiseptics containing quaternary ammonium compounds (Brooke, 2012; Kampmeier et al., 2017). Non-clinical multidrug resistant isolates of *S. maltophilia* have also been recovered from environments such as soil, water, plants and food sources (Berg et al., 2005; Qureshi et al., 2005; Brooke, 2012; Lin et al., 2017; Furlan and Stehling, 2018; Kim et al., 2018), and have the potential to cause community-acquired infections (Falagas et al., 2009; Chang et al., 2014).

High levels of innate antibiotic resistance in *S. maltophilia* isolates highlights the need for alternative treatment options to combat these infections. The use of bacteriophages, or phages, as viruses specifically lytic toward bacteria, is being investigated as a viable option. Phage therapy requires the isolation and characterization of phages prior to their use as prophylactic or therapeutic agents. Phage therapy holds several advantages over traditional antibiotics (Sulakvelidze et al., 2001; Burrowes et al., 2011; Golkar et al., 2014). First, phages possess a narrow host range, only infecting a few strains within a species, enabling precise targeting of the treatment, and preventing disruption of the patient’s normal microbiota. Second, phages are self-replicating, such that phage replication can significantly increase phage abundance at the site of the bacterial infection. Third, phages use a different mechanism of action to kill bacteria than chemical antibiotics, and so are virulent against antibiotic resistant bacteria. Fourth, few side effects have been reported during or after phage application, including immune responses, suggesting that higher organisms are adapted to the presence of phages within tissues or on body surfaces. In addition, the high abundance of phages in the environment make them relatively simple to isolate. As well, phages are relatively easy to manipulate through molecular approaches to create therapeutically enhanced phages which exhibit improved performance *in vivo*, and phage biology is becoming better understood such that the development of new therapeutic phages will progress more rapidly than it has in the past. Ideally, a therapeutic cocktail of multiple phages targeting several bacterial strains with different phage-receptors will be developed to increase the host range of the cocktail and protect against receptor-mutation mediated resistance (Burrowes et al., 2011; Golkar et al., 2014).

Twenty phages specific to *S. maltophilia* have been isolated and characterized to date. Eleven phages (Sm1, IME13, IME15, S3, Sm14, ΦSMA5, DLP1, DLP2, DLP4, DLP5, DLP6) have been isolated and characterized specifically for their therapeutic potential. For example, the *Myoviridae* phage ΦSMA5 has an extensive host range successfully infecting 61 out of 87 strains tested (Chang et al., 2005), while Sm1 is the first *S. maltophilia* phage used in a murine model where it was found to provide 100% protection against *S. maltophilia* infection. The broad host range of ΦSMA5 and significant *in vivo* protection Sm1 provides in a murine model are promising examples of therapeutic phages that can be used for the treatment of antibiotic resistant *S. maltophilia* infections.

## Materials and Methods

### Bacterial Strains and Growth Conditions

Initial phage isolation was accomplished with five clinical *S. maltophilia* strains (D1585, D1571, D1614, D1576, D1568) from the Canadian *Burkholderia cepacia* complex Research and Referral Repository (CBCCRRR; Vancouver, BC, Canada). An additional 22 *S. maltophilia* clinical isolates were obtained from the Provincial Laboratory for Public Health - North (Microbiology), Alberta Health Services for host range analysis. Strains SMDP92 and ATCC 13637 were gifted by Dr. Jorge Giron. Strains were grown aerobically at 30°C on Luria-Bertani solid media until single colonies were visible (16–36 h) or in LB broth with shaking at 225 RPM.

### Bacteriophage Isolation, Propagation, and Host Range

The phage DLP3 (vB_SmaS_DLP_3) was isolated from soil collected in Edmonton, Alberta, Canada using host strain *S. maltophilia* D1571. No plants were associated with the soil sample. Approximately 10 ml of soil was mixed with 10 ml of LB broth, 1 ml of modified suspension media (SM) (50 mM Tris-HCl pH 7.5, 100 mM NaCl, 10 mM MgSO4) and 100 μl of a D1571 overnight culture. The slurry was incubated overnight at 30°C with shaking at 225 RPM. The supernatant was filter-sterilized with a Millex-HA 0.45 μm syringe-driven filter (Millipore, Billerica, MA, United States) and stored at 4°C, as performed previously (Peters et al., 2015, 2017, 2019). A single plaque was picked to propagate a working-stock solution for analysis using top-agar overlays. Briefly, 100 μl of overnight D1571 culture and 100 μl DLP3 stock (∼10^9^ PFU/ml) were mixed and incubated for 5 min at room temperature then added to 3 ml of 0.7% LB top agar. The mixture was poured onto an LB plate and incubated for 18 h at 30°C. The top agar of plates showing confluent lysis was scraped into a 50 ml Falcon tube. A 3 ml aliquot of SM was added for each plate scraped and the slurry was shaken for 1 min followed by centrifugation (5 min at 10,000 × *g*) and filter sterilization. Host range analysis was performed on 29 *S. maltophilia* and 19 *Pseudomonas aeruginosa* strains using serially diluted DLP3 lysate into SM. A 10 μl aliquot of each concentration was spotted in triplicate onto a plate containing bacterial culture in a top-agar overlay and incubated overnight at 30°C.

### Electron Microscopy

Phage lysate for electron microscopy was prepared using LB plates and top agar made with agarose and filter sterilized using a 0.22 μm filter. A carbon-coated copper grid was overlaid with 10 μl of phage lysate for 2 min then stained with 4% uranyl acetate for 30 s. A Philips/FEI (Morgagni) transmission electron microscope (TEM) with charge-coupled device camera at 80 kV (University of Alberta Department of Biological Sciences Advanced Microscopy Facility) was used to obtain TEM images. The capsid diameter, tail length and tail width of ten virions were measured using ImageJ and averages calculated using Microsoft Excel.

### Phage DNA Isolation, Sequencing, and RFLP Analysis

Genomic DNA was isolated from a high-titer DLP3 stock (10^9^ PFU/ml). Lysate was clarified by spinning 10,000 × *g* for 10 min and the supernatant was subsequently treated with 100 μl 100x DNase I buffer (1 M Tris–HCl, 0.25 M MgCl2, 10 mM CaCl2), 10 μl DNase I (Thermo Scientific, Waltham, MA, United States), 6 μl RNase (Thermo Scientific) and incubated 1 h at 37°C. A 400 μl aliquot of 0.5 M EDTA (pH 8.0), SDS (final concentration of 2%) and Proteinase K (final concentration of 400 μg/ml) was added followed by incubation at 55°C overnight. A 1/2 volume of 6 M NaCl was added and the solution was vortexed at high speed for 30 s followed by centrifugation at 17,900 × *g* for 30 min. The supernatant was transferred to a fresh tube with an equal volume of 100% isopropanol and stored at −20°C for at least 1 h to overnight. The DNA was pelleted with centrifugation at 17,900 × *g* for 20 min at 4°C followed by three 70% ethanol washes. The pellet was dried at room temperature and resuspended in nuclease-free water. Purity and concentrations of eluted DNA were checked with a NanoDrop ND-1000 spectrophotometer (Thermo Scientific, Waltham, MA, United States). DLP3 genomic (gDNA) DNA was sequenced using both Illumina and Pacific Biosciences technologies. A Nextera XT library was generated for paired-end sequencing on MiSeq (Illumina) platform using MiSeq v2 reagent kit. Restriction fragment length polymorphism (RFLP) analysis was used with 15 FastDigest (Thermo Scientific) restriction enzymes: *Eco*RI, *Xba*I, *Bam*HI, *Hin*dIII, *Kpn*I, *Sma*I, *Sph*I, *Pst*I, *Sac*I, SaII, *Apa*I, *Cla*I, *Nde*I, *Spe*I, Xhol. Restriction reactions were set up using 1 μl FastDigest enzyme, 2 μl FastDigest restriction buffer, 1 μg of phage DNA and nuclease-free water to bring the final volume to 20 μl. Reactions were separated on a 0.8% (wt/vol) agarose gel in 1x TAE (pH 8.0).

### Bioinformatic Analysis of the DLP3 Genome

A 96,852 bp contig assembled from the Illumina reads with SPAdes 3.8.0 was identified for further analysis. No gaps or ambiguous sites were found in the assembly, which has a mean coverage of 114 reads and Q40 of 93.8%. Prediction of open reading frames (ORFs) was accomplished with the GLIMMER plugin (Delcher et al., 2007) for Geneious (Kearse et al., 2012) using the Bacteria and Archaea setting, as well as GeneMarkS for phage (Besemer et al., 2001). Conserved domain searches were performed using CD-Search with the CDD v3.16 – 50369 PSSMs database (Marchler-Bauer et al., 2011). Phyre (Kelley et al., 2015), HHblits (Remmert et al., 2011; Zimmermann et al., 2018) and ITASSER (Yang and Zhang, 2015) were used to gain insights into possible functions of hypothetical proteins or to provide more support for putative functions. BLASTn and BLASTp were used to gain information on relatives based on genomic data and individual proteins, respectively (Altschul et al., 1997). The NCBI non-redundant protein sequence and nucleotide collection databases (update dates for both: 2018/08/26) were used for the BLASTp and BLASTn searches, respectively. BLASTp results above 1.00E-03 were annotated as hypothetical proteins. tRNAs were identified using the general tRNA model with tRNAscan-SE software (Schattner et al., 2005).

### D1585 *pilT* Deletion Construction

A *pilT* clean deletion mutant of *S. maltophilia* D1585 was constructed using overlap-extension PCR and allele exchange as previously described (McCutcheon et al., 2018). Briefly, regions upstream and downstream of the *pilT* gene in D1585 were PCR amplified using Phusion High-Fidelity DNA Polymerase (New England Biolabs) with primer pairs UpF-*Hin*dIII (GGGC**AAGCTT**CAGTACCTGCGGCTTCACTG) and UpR-OE (*CTCGAACAGGCGCTTGGACGCTTTGTTCTT*TACGG) for the upstream region, and DnF-OE (*AAGAACAAAGCGTC CAAGCGCCTGTTCGAG*TAAGG) and DnR-*Xba*I (GGGC **TCTAGA**CTTCAGCTTGTGGATCTCGC) for the downstream region. Overlap regions are italicized and restriction enzyme recognition sites are bolded. Following overlap-extension PCR, the deletion cassette was ligated into pEX18Tc and this plasmid, pD1585Δ*pilT*, was transformed into *E. coli* S17-1 for bacterial mating with D1585. Single crossover transconjugants were selected on LB agar containing 100 μg/mL tetracycline and merodiploid status was confirmed by colony PCR with *pilT* specific primers pilTF (GTTCCGTTGAATCAGGAGGC) and pilTR (GAGGGCATGTACCAGGAAAC). Positive merodiploids were grown in LB broth and plated on LB with 10% sucrose to select for double crossover Δ*pilT* mutants that were confirmed by colony PCR as above. For complementation, the D1585 *pilT* gene was cloned into pBBR1MCS using pilTF and pilTR primers with tails for *Hin*dIII and *Xba*I restriction enzymes (Thermo Fisher) to create pD1585*pilT*.

### Phage Plaquing Assays

DLP3 plaquing ability on wildtype and mutant strains of D1585 and 280 was determined by spot assay on bacterial lawns as previously described (McCutcheon et al., 2018). Briefly, bacterial strains were grown in 1/2 LB supplemented with 35 μg/mL chloramphenicol at 30°C with aeration at 225 RPM for 18 h. 100 μL of overnight culture was used in a top agar overlay containing 35 μg/mL chloramphenicol and allowed to solidify. DLP3 lysate standardized to 10^10^ PFU/mL on *S. maltophilia* D1571 was ten-fold serially diluted in SM to 10^3^ PFU/mL and 5 μL of each dilution was spotted on the prepared plates in triplicate. Plates were incubated upright at 30°C and imaged after 16 h. Each experiment was repeated in biological and technical triplicate.

### Protein Isolation and Mass Spectrometry

Isolation of DLP3 protein for SDS-PAGE analysis was accomplished following a protocol for the formation of ghost particles (Boulanger, 2009). Briefly, sterile DLP3 lysate (∼1 × 10^9^) was clarified twice with 10,000 × *g* centrifugations and treated with nucleases following the DNA isolation protocol described above. After the incubation, an equal volume of 10 M LiCl was added and the solution was incubated at 46°C for 10 min, followed by 10-fold dilutions into sterile Milli-Q water. The released DLP3 gDNA was digested with an addition of 10 mM MgCl_2_ and 50 U of RNase-free DNaseI per 1 × 10^12^ PFU. This solution was incubated overnight at 37°C, followed by ultracentrifugation at 28,700 × *g* for 1.2 h. The supernatant was discarded and pellets were resuspended with 100 μl SM. An aliquot of the sample was diluted in half with 2x Laemmli sample buffer (10% [v/v] beta-mercaptoethanol [BME], 6% [w/v] SDS, 20% [v/v] glycerol, 0.2 mg/ml bromophenol blue) and incubated 10 min at 99°C.

An SDS-PAGE gel with a 4% stack and a 7.5% resolving portion was made with 40% 37.5:1 acrylamide/bis-acrylamide solution (Bio-Rad) and fresh 10% ammonium persulfate. The gel was loaded into a Mini-PROTEAN electrophoresis chamber (Bio-Rad) using 1x running buffer. A 6 μl aliquot of PageRuler Plus Prestained Protein Ladder (Thermo Scientific) was used as a molecular weight standard and 6–12 μl of DLP3 ghost particles in 1x sample buffer was loaded into the remaining wells. The gel was run at 180 kV for 75 min and placed in Coomassie R-250 stain for 1 h with gentle rocking. The gel was destained over 2 h, with the destaining solution replaced every 30 min. The gel was placed in a 50 ml Falcon tube with Milli-Q to transport the gel for mass spectrometry analysis at the Alberta Proteomics and Mass Spectrometry (APM) facility located at the University of Alberta.

In-gel trypsin digestion was performed on the samples. The lane was cut into seven equal gel sections, destained twice in 100 mM ammonium bicarbonate/acetonitrile (ACN) (50:50), reduced (10 mM BME–100 mM bicarbonate) and then alkylated (55 mM iodoacetamide–100 mM bicarbonate). After dehydration, trypsin digestion (6 ng/μl) was allowed to proceed overnight at room temperature. Tryptic peptides were extracted from the gel using 97% water–2% acetonitrile–1% formic acid followed by a second extraction using 50% of the initial extraction buffer and 50% acetonitrile. Fractions containing tryptic peptides were resolved and ionized using nanoflow high-performance liquid chromatography (HPLC) (Easy-nLC 1000; Thermo Scientific) coupled to a Q Exactive Orbitrap mass spectrometer (MS) (Thermo Scientific). Nanoflow chromatography and electrospray ionization were accomplished by using a Pico- Frit fused silica capillary column (ProteoPepII; C18) with a 100-μm inner diameter (New Objective) (300 Å, 5 μm pore size). Peptide mixtures were injected onto the column at a flow rate of 3,000 nl/min and resolved at 500 nl/min using 75-min linear gradients of 4% to 40% (vol/vol) aqueous ACN with 0.2% (vol/vol) formic acid. The mass spectrometer was operated in data-dependent acquisition mode, recording high-accuracy and high-resolution Orbitrap survey spectra using external mass calibration, with a resolution of 35,000 and m/z range of 400 to 2,000. The 15 most intensely multiply charged ions were sequentially fragmented by HCD fragmentation. After two fragmentations, all precursors selected for dissociation were dynamically excluded for 60 s. Data were processed using Proteome Discoverer 1.4 (Thermo Scientific). The UniProt *Stenotrophomonas* database and all DLP3 proteins were searched using SEQUEST (Thermo Scientific). Search parameters included a precursor mass tolerance of 10 ppm and a fragment mass tolerance of 0.8 Da. Peptides were searched with carbamidomethyl cysteine as a static modification and oxidized methionine and deamidated glutamine and asparagine as dynamic modifications.

### Determination of DLP3 Lifestyle

Top agar overlay plates showing confluent lysis of D1571 by DLP3 were used to obtain resistant colonies. Briefly, 3 ml of SM was added to the plates and a sterile glass rod was used to gently skim the agar. The SM was collected and placed into microcentrifuge tubes, then centrifuged at 5,000 × *g* for 5 min. The supernatant was discarded and 1 ml of fresh SM was added to resuspend the pellet, followed by centrifugation at 5,000 × *g* for 5 min. This wash step was repeated three times in total. Following the final wash centrifugation, the supernatant was removed and the pellet was resuspended in 500 μl LB broth. Cells were serially diluted with LB and plated on LB plates, then incubated at 30°C for 16 h. Single colony isolates were selected for further study and tested for superinfection resistance using overnight cultures of every isolate in a top agar overlay assay with DLP3. After an 18 h incubation at 30°C, the plates were observed for plaque development. Single colony isolates without plaque development were retained for analysis.

### Erm Functionality

Triplicate minimal inhibitory concentration (MIC) experiments on *S. maltophilia* strains D1571 and D1571:DLP3 were conducted using established protocols (Wiegand et al., 2008) to study the functionality of the DLP3-encoded Erm. Overnight cultures were grown at 30°C in 5 mL LB. A 1:100 subculture was grown at 30°C to an OD_600_ of 0.1 in Mueller-Hinton broth (MH) and used in 96 well plates containing an erythromycin dilution series (MP Biomedicals). Following a 16 h incubation, OD_600_ absorbance was obtained using a Wallac 1420 VICTOR2 multilabel counter (PerkinElmer, Waltham, MA, United States) and values were averaged using Excel. Statistical analysis was conducted using GraphPad Prism 7 (GraphPad Software Inc., San Diego, CA, United States) to perform a two-way analysis of variance (ANOVA) with Sidak’s multiple comparisons.

### Growth Analysis of Wild Type D1571 and the DLP3 Lysogen

Single colony triplicate overnight cultures of wild type D1571 and the lysogen D1571:DLP3 were grown in LB broth at 30°C with shaking. Subcultures (1:100) for each sample were performed using LB broth and each subculture was grown to an OD_600_ of ∼0.32 at 30°C with 225 RPM shaking. Subcultures were distributed in triplicate aliquots of 200 μl in 96 well plates; an LB broth control was included for each plate. The OD_600_ was then obtained for each plate using a Wallac 1420 VICTOR^2^ multilabel counter (PerkinElmer, Waltham, MA) at the following time points: 0, 2, 4, 6, 8 h. The OD_600_ data were used to determine the growth rate (μ) with the established formula: log_10_ *N* − log_10_ *N*_0_ = (μ/2.303) (*t* − *t*_0_), whereby *N*_0_ is the time zero (*t*_0_) OD_600_ reading and *N* is the final OD_600_ reading obtained at a specific time (*t*) in the experiment. Resulting data were analyzed with GraphPad Prism 7 (GraphPad Software Inc., San Diego, CA, United States) to graph the growth curve and growth rate. Statistical analysis of the growth curve and growth rate data were performed in GraphPad Prism 8 using two-way repeated measurement ANOVA with multiple comparisons (Sidak’s).

### *Galleria mellonella* Killing and Phage Rescue Assays

*G. mellonella* infections were performed as previously described, with modifications (Seed and Dennis, 2008; Kamal et al., 2019). Single colony triplicate overnight cultures of wildtype strain D1571 and the DLP3 lysogen, D1571:DLP3, were grown aerobically at 37°C in LB for 19 h corresponding to approximately 2 × 10^10^CFU/mL. Cultures were standardized by OD_600_, washed once in 1× phosphate-buffered saline (PBS, pH 7.4) and serially diluted tenfold in PBS. *G. mellonella* larvae were bred in-house at 30°C using artificial food (wheat germ: 264 g, brewer’s yeast: 132 g, beeswax: 210 g, glycerol: 132 g, honey: 132 g, water: 66 g) and larvae weighing approximately 250 mg were selected for experiments. Each experiment consisted of ten larvae per group and 5 μL aliquots of bacterial culture were injected into the rear left proleg of each larva using a 250 μL Hamilton syringe fitted with a repeating dispenser. Sterile PBS injected larvae were used as negative controls and showed 100% survival for the duration of the experiment. Colony counts on LB agar were used to determine the CFUs injected. Following injection, larvae were placed in a static incubator in the dark at 37°C and scored for death every 24 h until 120 h post-infection (hpi). Larvae were considered dead when they did not respond to touch with movement.

For DLP3 phage rescue trials, wildtype D1571 culture was prepared as described above and an inoculum of approximately 8 × 10^6^CFU/larvae was chosen. DLP3 lysate was used at 8.9 × 10^10^ PFU/mL. Larvae were injected with 10 μL of DLP3 lysate dilutions to give MOIs of approximately 100 and 50 into the rear right proleg 1.5 hpi with D1571. Aliquots of 5 μL PBS and 10 μL SM were used in place of bacteria and phage, respectively, for negative controls. Each worm was therefore injected with 15 μL total volume. Larvae were incubated and scored for survival as above. Results from three separate trials were combined and survival at each timepoint was plotted using the Kaplan–Meier method with error bars for standard error using GraphPad Prism 8. Statistical analysis of survival differences was completed using the Log-rank (Mantel–Cox) test.

## Results and Discussion

### Isolation, Morphology, Host Range, and RFLP Analysis

**FIGURE 1:**
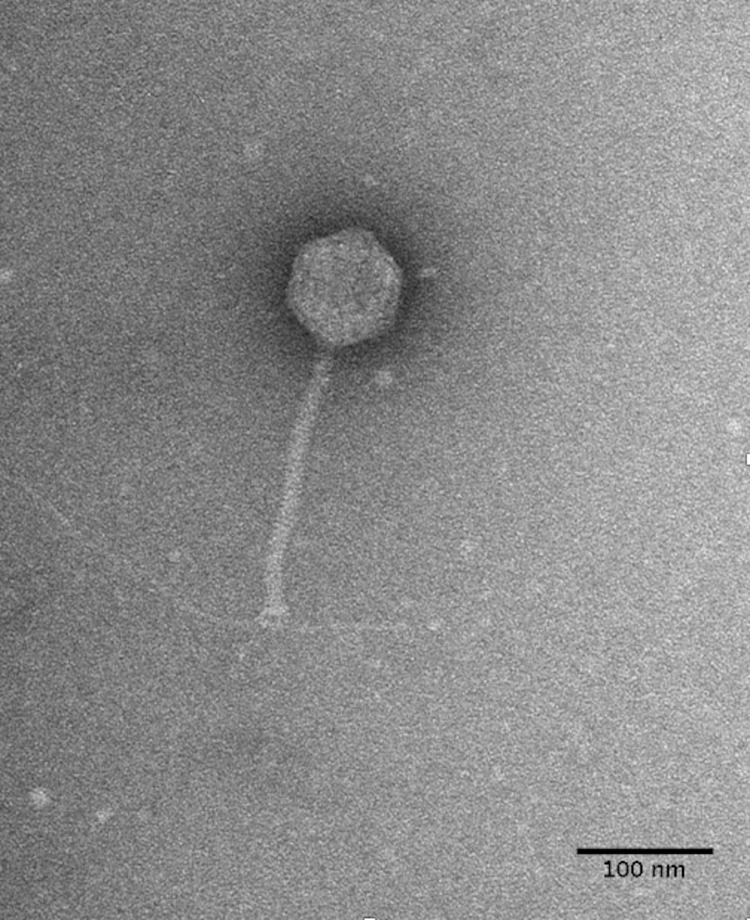
DLP3 *Siphoviridae* morphology. Phage lysate was applied to a carbon-coated copper grid and stained with 4% uranyl acetate. Transmission electron micrographs were obtained at 180,000× magnification. A *S. maltophilia* D1571 pili is shown to be interacting with the baseplate portion of the DLP3 tail. The averaged measurements for tail length, capsid length and width from ten virions is 202, 92, and 84 nm, respectively.

Bacteriophage DLP3 (vB_SmaS-DLP_3) was isolated from soil using clinical *S. maltophilia* strain D1571. Transmission electron microscopy (TEM) enabled the classification of DLP3 as a *Siphoviridae* of the B1 morphotype (Ackermann and Eisenstark, 1974) due to the long, non-contractile tail averaging 202.2 ± 5.7 nm and isometric capsid with a length and width of 92.8 ± 4.1 and 84.0 ± 2.8 nm, respectively (Figure 1). No tail fibers were observed in the TEM images. The host range of DLP3 against all 29 clinical *S. maltophilia* isolates reveals a broad tropism through the successful infection of 22 strains, although DLP3 is not capable of infecting the *P. aeruginosa* strains tested (Table 1). The restriction fragment length polymorphism analysis revealed DLP3 genomic DNA is resistant to the 15 restriction enzymes screened. These results suggest DLP3 contains modified DNA, although the exact types of modifications are currently undetermined.

**TABLE 1:** Host range analysis for *S. maltophilia* bacteriophage DLP3 on *S. maltophilia* and *P. aeruginosa* strains.

*S. maltophilia* strain	Efficiency of plating
101	++
102^*b*^	+++
103	++
152	−
155	++++
174	++
176	++++
213	++
214	++
217	−
218	+
219	+++
230	++++
236	+
242	++
249^*b*^	+
278	−
280	+++
282	+
287	++
446	−
667	+
D1585^a^	+++
D1571^a,b^	++++
D1614^a,b^	−
D1576^a,b^	+++
D1568^a^	−
SMDP92^b^	++++
ATCC13637	−
***P. aeruginosa* strain**	
PA01	−
HER1004	−
HER1012	−
14715	−
Utah3	−
Utah4	−
14655	−
6106	−
pSHU-OTE	−
D1606D^a^	−
D1615C^a^	−
D1619M^a^	−
D1620E^a^	−
D1623C^a^	−
ENV003^a^	−
ENV009^a^	−
FC0507^a^	−
R285	−
14672	−

*−, no sensitivity to phage; +, plaques at 10–2; ++, clearing at 10–2; +++, plaques at 10–4; ++++, plaques at 10–6. ^*a*^Isolates from the Canadian *Burkholderia cepacia* complex Research Referral Repository. ^*b*^Strain susceptible to phage DLP5.*

### Genomic Characterization

**FIGURE 2:**
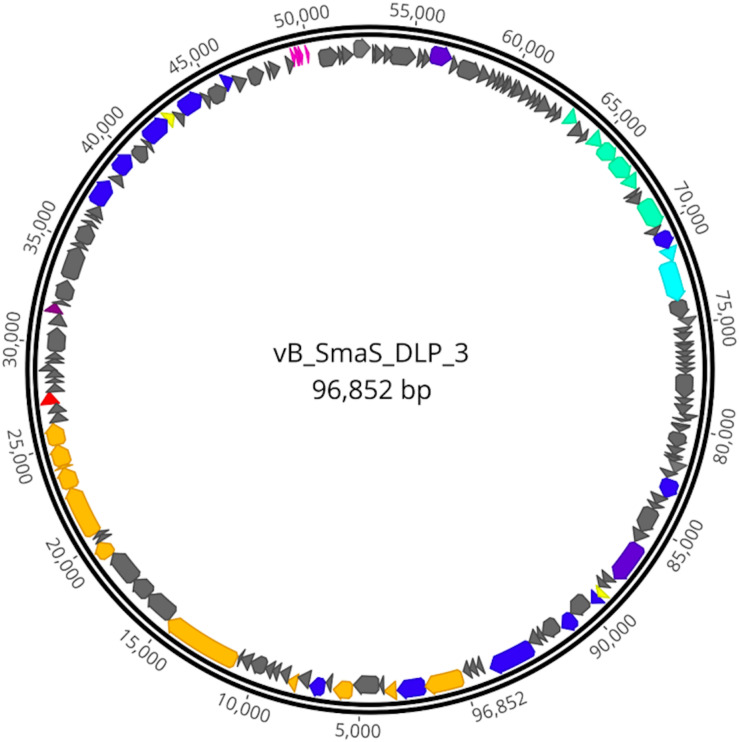
Genome map of DLP3. Scale in bp is shown on the outer periphery. Predicted functions are grouped by color: teal; moron, gray; hypothetical, light blue; DNA packaging, pink; tRNA, red; lysis, green; virion morphogenesis, dark blue; DNA replication and repair, purple; auxiliary metabolism, and yellow; regulatory.

The DLP3 genome is 96,852 bp long with a 58.3% global GC content. No low coverage or ambiguous regions were identified with the assembled contig, which has a mean coverage of 114 and a Q40 of 99.6%. NCBI non-redundant protein sequence database searches indicate that DLP3 shares a high identity to the *Siphoviridae* phage vB_SmaS_DLP_5 (DLP5) (Peters and Dennis, 2018). DLP5 is the type species of the new genus *Delepquintavirus* and based on the genomic similarities between DLP3 and DLP5, DLP3 is also a member of this new genus. Open reading frame calling with Glimmer and GeneMarkS identified a total of 148 protein coding domain sequences (CDS) covering 95% of the genome (Figure 2 and Table 2). DLP3 also encodes five tRNA genes with different specificities: Tyr (GTA), Sup (CTA), Ser (GCT), Ile (GAT), Glu (TTC). A total of 97 proteins could not be assigned functions due to lack of significant results from both BLASTp and CD-Search. The DLP3 genome with putative gene annotations has been deposited in GenBank under accession number MT110073.

**TABLE 2:** Genome annotations for DLP3 obtained from BLASTp and CD-Search data.

CDS	Interval	Length (AA)	Putative function	Species	Coverage (%)	*E*-Value	Identity (%)	Accession
1	48–1856	602	Portal protein	DLP5^a^	98	0	83	ATS92275.1
2	1856–3193	445	ParB-like nuclease domain protein	DLP5	100	0	75	ATS92281.1
3	3249–3854	201	Serine protease	DLP5	98	1.00E-113	80	ATS92325.1
4	3847–4017	56	Hypothetical protein gp_005	DLP5	100	3.00E-24	77	ATS92409.1
5	4090–5307	405	Hypothetical protein gp_006	DLP5	100	0	79	ATS92283.1
6	5340–6272	310	Major capsid protein	DLP5	100	0	93	ATS92299.1
7	6358–6624	88	Hypothetical protein gp_008	DLP5	100	3.00E-33	65	ATS92383.1
8	6693–7436	247	Ribonuclease E	DLP5	100	3.00E-113	73	ATS92315.1
9	7457–8068	203	Hypothetical protein gp_010	DLP5	100	2.00E-121	82	ATS92324.1
10	8068–8493	141	Tail protein	DLP5	99	6.00E-71	77	ATS92347.1
11	8490–8936	148	Hypothetical protein gp_012	DLP5	99	3.00E-82	76	ATS92342.1
12	9012–9305	97	Hypothetical protein gp_013	DLP5	100	5.00E-50	85	ATS92376.1
13	9316–9693	125	Hypothetical protein gp_014	DLP5	100	4.00E-48	67	ATS92360.1
14	9693–10448	251	Hypothetical protein gp_015	DLP5	100	2.00E-152	82	ATS92314.1
15	10465–10959	164	Hypothetical protein gp_016	DLP5	100	9.00E-83	81	ATS92333.1
16	11016–11144	42	Hypothetical protein gp_017	DLP5	95	2.00E-15	83	ATS92415.1
17	11151–14915	1254	Tape measure protein	DLP5	99	0	83	ATS92270.1
18	14917–16500	527	Hypothetical protein gp_019	DLP5	100	0	79	ATS92277.1
19	16500–17474	324	Hypothetical protein gp_020	DLP5	100	0	83	ATS92295.1
20	17474–19153	559	Hypothetical protein gp_021	DLP5	100	0	74	ATS92276.1
21	19150–19968	272	Minor tail protein	DLP5	100	0	90	ATS92307.1
22	19968–20255	95	Hypothetical protein gp_023	DLP5	100	5.00E-62	98	ATS92377.1
23	20252–20455	67	Hypothetical protein gp_024	DLP5	100	1.00E-38	93	ATS92399.1
24	20445–22925	826	Tail protein	DLP5	100	0	86	ATS92271.1
25	22925–23923	332	Tail assembly protein	DLP5	100	0	86	ATS92294.1
26	23926–24102	58	Tail assembly protein	DLP5	100	3.00E-30	88	ATS92406.1
27	24110–25081	323	Tail assembly protein	DLP5	100	2.00E-169	70	ATS92296.1
28	25085–26107	340	Tail protein	Salvo^b^	100	3.00E-76	43	AHB12239.1
29	26183–26611	142	Hypothetical protein gp_030	DLP5	100	1.00E-91	93	ATS92345.1
30	26611–27096	161	Hypothetical protein gp_031	DLP5	100	6.00E-87	91	ATS92334.1
31	27096–27653	185	Lysozyme	DLP5	100	2.00E-120	89	ATS92330.1
32	27655–28059	134	Hypothetical protein gp_033	DLP5	100	3.00E-80	88	ATS92352.1
33	28110–28280	56	Hypothetical protein gp_034	DLP5	78	1.00E-11	70	ATS92408.1
34	28329–28715	128	DUF2500 containing protein	DLP5	100	3.00E-77	89	ATS92350.1
35	28687–28974	95	Hypothetical protein gp_036	DLP5	93	2.00E-57	96	ATS92380.1
36	29077–29271	64	Hypothetical protein					
37	29275–29574	99	Hypothetical protein gp_041	DLP5	95	2.00E-58	95	ATS92374.1
38	29634–30791	385	Hypothetical protein gp_042	DLP5	100	0	78	ATS92287.1
39	30898–31506	202	Hypothetical protein gp_043	DLP5	87	2.00E-82	70	ATS92323.1
40	31499–31951	150	Phosphoglycerate kinase	DLP5	100	2.00E-103	96	ATS92341.1
41	31951–32136	61	Hypothetical protein gp_046	DLP5	100	2.00E-25	79	ATS92401.1
42	32197–33141	314	Hypothetical protein gp_047	DLP5	100	3.00E-138	85	ATS92300.1
43	33255–34856	533	Hypothetical protein gp_048	DLP5	98	2.00E-156	50	ATS92279.1
44	34856–35137	93	Hypothetical protein gp_049	DLP5	100	5.00E-35	64	ATS92378.1
45	35139–36056	305	Hypothetical protein gp_050	DLP5	100	0	90	ATS92301.1
46	36060–36251	63	Hypothetical protein	BCC^c^	98	5.00E-08	46	WP_046196969.1
47	36389–36571	60	Hypothetical protein gp_052	DLP5	85	1.00E-19	78	ATS92407.1
48	36568–37128	186	Hypothetical protein gp_053	DLP5	100	5.00E-128	93	ATS92329.1
49	37125–38414	429	Helicase	DLP5	100	0	90	ATS92282.1
50	38481–38981	166	Hypothetical protein gp_055	DLP5	100	2.00E-86	80	ATS92331.1
51	38974–39957	327	Primase	DLP5	100	0	77	ATS92297.1
52	40036–40812	258	Hypothetical protein gp_057	DLP5	100	3.00E-157	89	ATS92312.1
53	40860–41075	71	Hypothetical protein gp_058	DLP5	100	4.00E-41	92	ATS92395.1
54	41072–42430	452	Superfamily II DNA or RNA helicase	DLP5	100	0	90	ATS92280.1
55	42427–42828	133	Transcriptional regulator	DLP5	100	6.00E-68	74	ATS92353.1
56	42831–43208	125	Hypothetical protein gp_061	DLP5	100	1.00E-62	81	ATS92362.1
57	43208–44425	405	RecA	DLP5	100	0	90	ATS92285.1
58	44425–44799	124	Hypothetical protein gp_063	DLP5	100	9.00E-83	94	ATS92359.1
59	44799–45680	293	Hypothetical protein gp_064	DLP5	100	0	87	ATS92304.1
60	45677–46174	165	RuvC	DLP5	99	5.00E-111	93	ATS92332.1
61	46152–46766	204	Hypothetical protein gp_066	DLP5	100	5.00E-108	78	ATS92321.1
62	46848–47657	269	Hypothetical protein gp_067	DLP5	96	2.00E-153	83	ATS92308.1
63	47840–47947	35	Hypothetical protein gp_069	DLP5	100	5.00E-13	94	ATS92417.1
64	48028–48504	158	Hypothetical protein gp_070	DLP5	100	3.00E-96	91	ATS92336.1
65	48839–49174	111	Hypothetical protein gp_071	DLP5	100	8.00E-60	82	ATS92357.1
66	50468–51409	313	Hypothetical protein gp_073	DLP5	100	6.00E-127	61	ATS92313.1
67	51397–51585	62	Hypothetical protein					
68	51582–52145	187	Hypothetical protein gp_074	DLP5	100	2.00E-124	91	ATS92328.1
69	52165–52959	264	SPFH domain-containing protein	DLP5	100	0	96	ATS92309.1
70	53058–53210	50	Hypothetical proteins					
71	53207–53653	148	Hypothetical protein gp_077	DLP5	100	3.00E-87	87	ATS92343.1
72	53650–54000	116	Hypothetical protein gp_078	DLP5	100	7.00E-74	90	ATS92365.1
73	53993–55207	404	Hypothetical protein gp_079	DLP5	100	7.00E-96	44	ATS92288.1
74	55279–55548	113	Hypothetical protein gp_081	DLP5	100	1.00E-65	84	ATS92368.1
75	55558–55899	89	Hypothetical protein gp_080	DLP5	98	8.00E-37	66	ATS92369.1
76	55887–56930	347	UDP-glucose 4-epimerase	DLP5	100	0	83	ATS92289.1
77	56962–57285	107	Hypothetical protein gp_083	DLP5	99	9.00E-56	78	ATS92370.1
78	57355–58425	356	Hypothetical protein gp_084	DLP5	100	0	73	ATS92290.1
79	58418–58939	173	Hypothetical protein gp_087	DLP5	45	6.00E-35	73	ATS92388.1
80	58936–59283	115	Hypothetical protein gp_086	DLP5	86	1.00E-36	64	ATS92361.1
81	59280–59456	58	Hypothetical protein gp_088	DLP5	100	4.00E-17	62	ATS92405.1
82	59465–59737	90	Hypothetical protein BV378_14040	*Nostoc* sp. RF31Y	97	8.00E-19	50	OUL25853.1
83	59737–59862	41	Hypothetical protein					
84	59859–60128	89	Hypothetical protein					
85	60132–60314	60	Hypothetical protein gp_089	DLP5	100	2.00E-05	53	ATS92403.1
86	60318–60737	139	Hypothetical protein gp_090	DLP5	84	3.00E-64	79	ATS92363.1
87	60734–61210	158	Hypothetical protein gp_091	DLP5	98	7.00E-60	62	ATS92339.1
88	61207–61458	83	Hypothetical protein gp_092	DLP5	100	1.00E-22	46	ATS92382.1
89	61455–61709	84	Hypothetical protein					
90	61709–62359	216	Hypothetical protein gp_093	DLP5	100	3.00E-100	65	ATS92319.1
91	62362–62649	95	Hypothetical protein gp_094	DLP5	96	4.00E-35	66	ATS92381.1
92	62646–62885	79	Hypothetical protein gp_095	DLP5	100	4.00E-31	70	ATS92392.1
93	63161–63688	175	Rhomboid membrane protein	DLP5	100	9.00E-96	78	ATS92322.1
94	63746–64342	198	Hypothetical protein gp_097	DLP5	98	8.00E-108	82	ATS92326.1
95	64339–64593	84	Hypothetical protein gp_098	DLP5	96	3.00E-31	68	ATS92385.1
96	64670–65308	212	PIG-L family deacetylase	DLP5	100	6.00E-114	75	ATS92320.1
97	65311–66252	313	WcaG	DLP5	100	0	95	ATS92298.1
98	66252–67298	348	WecE	DLP5	100	0	87	ATS92291.1
99	67295–67975	226	Methyltransferase	DLP5	100	1.00E-139	82	ATS92317.1
100	68043–68228	61	Hypothetical protein gp_103	DLP5	98	5.00E-20	62	ATS92400.1
101	68225–68647	140	Hypothetical protein gp_104	DLP5	97	8.00E-56	68	ATS92346.1
102	68647–70095	482	*N*-acetyl-alpha-D-glucosaminyl l-malate synthase	DLP5	100	0	93	ATS92278.1
103	70092–70403	103	Hypothetical protein gp_106	DLP5	100	1.00E-36	59	ATS92372.1
104	70375–71229	284	ParBc	DLP5	99	0	89	ATS92305.1
105	71229–71879	216	Hypothetical protein gp_108	DLP5	99	5.00E-125	85	ATS92318.1
106	71842–73755	637	Terminase large subunit	DLP5	100	0	91	ATS92274.1
107	73767–74660	297	Hypothetical protein gp_110	DLP5	100	0	95	ATS92303.1
108	74657–75067	136	DUF3310 containing protein	DLP5	100	1.00E-61	71	ATS92351.1
109	75129–75371	80	Hypothetical protein gp_112	DLP5	100	8.00E-37	74	ATS92390.1
110	75373–75765	130	Hypothetical protein gp_113	DLP5	92	7.00E-12	34	ATS92355.1
111	75841–75939	32	Hypothetical protein gp_114	DLP5	100	7.00E-09	81	ATS92418.1
112	75936–76253	105	Hypothetical protein gp_115	DLP5	96	6.00E-54	81	ATS92371.1
113	76250–76504	84	Hypothetical protein gp_116	DLP5	100	2.00E-42	79	ATS92387.1
114	76494–76859	121	Hypothetical protein gp_117	DLP5	98	6.00E-59	80	ATS92358.1
115	76859–77011	50	Hypothetical protein gp_118	DLP5	100	1.00E-22	88	ATS92412.1
116	77087–77335	82	Hypothetical protein gp_119	DLP5	100	2.00E-43	82	ATS92389.1
117	77389–78528	379	Hypothetical protein gp_120	DLP5	70	2.00E-143	94	ATS92286.1
118	78528–78878	116	Hypothetical protein gp_121	DLP5	99	4.00E-71	90	ATS92364.1
119	78904–79365	153	Hypothetical protein gp_122	DLP5	100	7.00E-78	75	ATS92340.1
120	79331–79561	76	Hypothetical protein gp_123	DLP5	100	3.00E-33	76	ATS92393.1
121	79558–79731	57	Hypothetical protein gp_124	DLP5	96	6.00E-23	78	ATS92404.1
122	79804–79920	38	Hypothetical protein					
123	79920–80228	102	Hypothetical protein gp_125	DLP5	100	3.00E-59	87	ATS92373.1
124	80254–80988	244	Hypothetical protein gp_126	DLP5	100	8.00E-153	83	ATS92316.1
125	80985–81350	121	Hypothetical protein gp_127	DLP5	96	1.00E-64	79	ATS92356.1
126	81347–81523	58	Hypothetical protein					
127	81516–81698	60	Hypothetical protein gp_128	DLP5	96	2.00E-29	86	ATS92402.1
128	81698–82180	160	DUF1643 containing protein	DLP5	100	2.00E-88	80	ATS92337.1
129	82180–82515	111	Hypothetical protein gp_130	DLP5	90	2.00E-44	83	ATS92367.1
130	82589–83485	298	DNA ligase	DLP5	100	0	92	ATS92302.1
131	83482–83895	137	Hypothetical protein gp_133	DLP5	100	4.00E-65	74	ATS92349.1
132	83895–84197	100	Hypothetical protein gp_134	DLP5	97	2.00E-47	77	ATS92375.1
133	84248–85459	403	Hypothetical protein gp_135	DLP5	100	0	81	ATS92284.1
134	85456–86028	190	Hypothetical protein gp_136	DLP5	100	3.00E-119	85	ATS92327.1
135	86122–88065	647	Pyruvate phosphate dikinase	DLP5	100	0	84	ATS92273.1
136	88141–88527	128	Hypothetical protein gp_138	DLP5	98	7.00E-76	90	ATS92348.1
137	88527–88868	113	Hypothetical protein gp_139	DLP5	100	5.00E-63	86	ATS92366.1
138	88879–89238	119	Transcriptional repressor	DLP5	95	3.00E-71	88	ATS92354.1
139	89228–89698	156	Tyrosine phosphatase family protein	DLP5	99	5.00E-93	86	ATS92338.1
140	89740–90759	339	Hypothetical protein gp_142	DLP5	98	0	77	ATS92292.1
141	90756–91532	258	Thymidylate synthase	DLP5	98	6.00E-121	72	ATS92311.1
142	91618–92493	291	Hypothetical protein gp_144	DLP5	97	1.00E-124	65	ATS92306.1
143	92483–92737	84	Hypothetical protein gp_145	DLP5	97	3.00E-35	71	ATS92386.1
144	92734–93231	165	Hypothetical protein gp_146	DLP5	96	1.00E-83	77	ATS92335.1
145	93260–95482	740	DNA polymerase I	DLP5	100	0	85	ATS92272.1
146	95794–96030	78	Hypothetical protein					
147	96134–96415	93	Hypothetical protein gp_149	DLP5	96	6.00E-52	87	ATS92379.1
148	96412–96741	109	Hypothetical protein gp_150	DLP5	100	2.00E-61	85	ATS92310.1

*Results below 0.01 were not included and the function was annotated as hypothetical. ^*a*^*Stenotrophomonas* phage DLP5, ^*b*^*Xylella* phage Salvo, and ^*c*^*Burkholderia cepacia* complex.*

The CD-Search did yield 37 DLP3 proteins with conserved domains predicted (Table 3). Three of the domains identified are domains of unknown function (DUF) which tended to be distributed throughout Gram-negative bacteria and to a lesser extent in Gram-positive bacteria, according to the species distribution for each DUF in pfam (Finn et al., 2014). There are six conserved domains (CD) identified which are involved in virion morphogenesis: phage portal protein superfamily, gp1; phage capsid family, gp6; phage tail proteins, gp10 and gp24; laminin G, gp28; and tape measure protein domain, gp17. Nine proteins with domains involved in DNA replication and repair identified with the CD-Search include two ParB domains, gp2 and 104; two helicases, gp49 and 54; Holliday junction resolvase, gp60; RecA recombinase, gp57; topoisomerase primase, gp51; DNA ligase, gp130; and DNA polymerase A, gp145. The remaining CD results appear to be quite diverse in their functions such as the SpoVK family domain involved in sporulation (gp38) (Fan et al., 1992), protein-tyrosine phosphatases (gp139 and gp141) typically involved in signal transduction (Whitmore and Lamont, 2012), and a membrane-associated serine protease of the rhomboid family (gp93). One CD identified that is of particular interest is the glycosyltransferase domain of gp102. T-even bacteriophages have been shown to use glycosyltransferases for DNA modification by linking a glycosyl group to hydroxymethyl-cytosine, thus protecting the DNA against digestion by bacterial restriction systems (Bair and Black, 2007). Another role for glycosyltransferases within bacteriophages is highlighted by some *Shigella* phages which have been shown to seroconvert their host by modifying the *O*-antigen polysaccharides to prevent infection of the bacteria by other O-antigen receptor phages (Allison and Verma, 2000). The specific glycosyltransferase family is RfaB, a protein involved in the assembly of the LPS core of *Escherichia coli* K-12 (Pradel et al., 1992). This result suggests DLP3 may use the glycosyltransferase to modify the host LPS, similar to the seroconverting *Shigella* phages, though this has yet to be confirmed experimentally.

**TABLE 3:** The conserved domains found in the 148 DLP3 gene products.

Gp	Hit type	PSSM-ID	Interval	*E*-Value	Accession	Short name	Superfamily
1	Superfamily	327517	29–471	2.89E-49	cl19194	Phage_portal superfamily	–
2	Specific	214678	359–441	8.46E-06	smart00470	ParB	cl02129
3	Superfamily	317012	5–114	7.39E-23	cl24270	Peptidase_S78_2 superfamily	–
6	Superfamily	331903	20–307	5.06E-12	cl27082	Phage_capsid superfamily	–
10	Superfamily	321796	6–137	7.98E-15	cl02089	Phage_tail_S superfamily	–
17	Superfamily	331332	525–930	5.90E-15	cl26511	Neuromodulin_N superfamily	–
17	Superfamily	333387	163–230	3.22E-03	cl28567	HI1514 superfamily	–
19	Superfamily	316645	14–69	9.95E-03	cl16644	DUF4302 superfamily	–
21	Superfamily	312753	188–264	8.54E-16	cl10710	Phage_BR0599 superfamily	–
21	Superfamily	331404	18–261	5.62E-12	cl26583	DUF2163 superfamily	–
24	Specific	316107	207–368	2.18E-13	pfam13550	Phage-tail_3	cl26145
28	Superfamily	328935	62–184	1.13E-05	cl22861	LamG superfamily	–
31	Superfamily	331815	6–185	4.73E-34	cl26994	Glyco_hydro_108 superfamily	–
38	Superfamily	332389	92–371	4.11E-42	cl27568	TIP49 superfamily	–
41	Superfamily	332243	13–46	5.11E-03	cl27422	SecD superfamily	–
49	Superfamily	333705	167–405	5.02E-22	cl28885	RecA-like_NTPases superfamily	–
51	Superfamily	331610	31–322	7.81E-12	cl26789	Toprim_N superfamily	–
54	Superfamily	331760	51–451	4.03E-38	cl26939	DEXDc superfamily	–
55	Superfamily	322007	53–98	3.22E-04	cl02600	HTH_MerR-SF superfamily	–
57	Superfamily	333705	77–272	2.57E-48	cl28885	RecA-like_NTPases superfamily	–
60	Superfamily	328743	1–140	6.73E-14	cl21482	RuvC_resolvase superfamily	–
69	Specific	307341	23–207	2.15E-17	pfam01145	Band_7	cl19107
76	Specific	224012	1–336	1.19E-76	COG1087	GalE	cl21454
78	Superfamily	330522	59–126	2.19E-04	cl25701	RuvB_N superfamily	–
93	Superfamily	328780	29–159	3.68E-14	cl21536	Rhomboid superfamily	–
96	Specific	308281	5–122	3.46E-11	pfam02585	PIG-L	cl00929
97	Specific	223528	1–297	7.51E-40	COG0451	WcaG	cl25660
98	Superfamily	327488	20–340	5.46E-56	cl18945	AAT_I superfamily	–
99	Superfamily	327401	30–135	1.43E-04	cl17173	AdoMet_MTases superfamily	–
102	Specific	223515	1–323	4.33E-03	COG0438	RfaB	cl28208
104	Specific	214678	18–109	7.28E-17	smart00470	ParB	cl02129
104	Specific	224392	34–187	5.36E-08	COG1475	Spo0J	cl26722
108	Superfamily	314594	17–68	1.07E-11	cl13237	DUF3310 superfamily	–
128	Specific	311648	12–146	4.16E-44	pfam07799	DUF1643	cl01787
130	Superfamily	325160	25–186	2.14E-25	cl12015	Adenylation_DNA_ligase_like superfamily	–
130	Superfamily	330238	112–270	1.28E-08	cl25417	CDC9 superfamily	–
133	Superfamily	332389	206–341	8.78E-33	cl27568	TIP49 superfamily	–
133	Superfamily	332204	43–150	8.52E-03	cl27383	ERCC4 superfamily	–
135	Superfamily	331842	16–471	0.00E+00	cl27021	PtsP superfamily	–
139	Superfamily	330819	39–125	1.50E-10	cl25998	CDC14 superfamily	–
141	Superfamily	330819	85–189	1.80E-05	cl25998	CDC14 superfamily	–
145	Superfamily	322025	107–710	7.76E-64	cl02626	DNA_pol_A superfamily	–

### Phage DLP3 Relatedness to Phage DLP5

**FIGURE 3:**
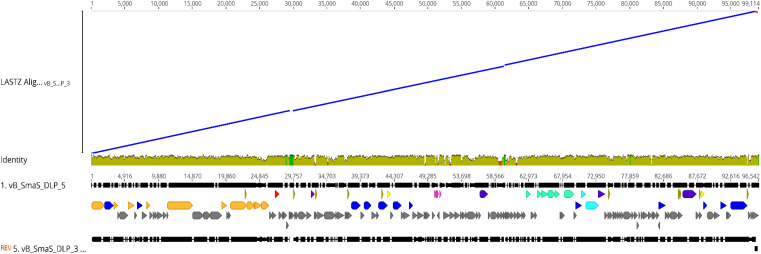
Genomic alignment of DLP3 to DLP5 using the Large-Scale Genome Alignment Tool. Identity is indicated by color: mustard yellow; >30%, green; deletion in DLP3, red; deletion in DLP5.

While characterizing the *Siphoviridae* phage DLP3 genome, it was evident through BLASTn and BLASTp searches that phages DLP3 and DLP5 are closely related. The genome size of DLP3 and DLP5 are similar (96,852 versus 96,542 bp), as is their GC content (58.3 versus 58.4%). BLASTn analysis of DLP3 against DLP5 revealed 81% identity over 90% of the DLP5 genome (0.0 *E*-value). A LASTZ alignment (Harris, 2007) of the phages using DLP5 as the reference sequence shows sequence identity >30% between their genomes, represented as a mustard yellow color in the consensus (Figure 3). BLASTn alignment between DLP3 to DLP5 gives a query coverage of 89% and 81% identity. Two small stretches with a breakdown in identity are observed between the DLP5 and DLP3 genomes, which is viewed as a breakdown in the alignment blocks of the dot plot around 30,000 and 60,000 bp (Figure 3). These stretches correspond to four genes (DLP05_037 to DLP05_039, plus DLP05_087) encoding hypothetical proteins with no significant matches in the NCBI database, no conserved domains, and no significant results when using structural prediction software such as HHpred and Phyre. Two additional genes, DLP05_045 and DLP05_068, which encode hypothetical proteins, are not present in the DLP3 genome. The DLP05_045 gene product shares 93% coverage with 36% identity (1.0E-08) to a *Polaromonas naphthalenivorans* hypothetical protein which contains no conserved domains, whereas the DLP05_068 gene product did not have similar sequences in the NCBI database or conserved domains predicted. Both phages encode five tRNAs, with four of the five tRNAs sharing the same specificity: Sup-CTA, Glu-TTC, Ser-GCT, Tyr-GTA. Phage DLP3 differs from DLP5 with respect to the fifth tRNA, which is Ile-GAT in DLP3 but Gly-TCC in DLP5. The amino acid usage of each phage does not explain the differences, as they each have the same usage rates for isoleucine (4.7%) and glycine (7.7%), but there are several nucleotide changes observed in this region when comparing DLP3 to DLP5.

Besides the genomic similarities observed between DLP3 and DLP5, they also share morphological similarities. The DLP3 and DLP5 phages have the same measurement averages for their head width (84 nm) and length (92 nm), as well as tail length (202 nm). The host range of DLP3 versus DLP5 is significantly different, with DLP3 exhibiting a broad host range infecting 22 *S. maltophilia* strains, whereas DLP5 only infects six (Table 1). Of the six strains DLP5 is capable of infecting (102, 249, D1571, D1614, D1576, SMDP92), DLP3 is only unable to infect the DLP5 host strain D1614. The virion morphogenesis proteins of DLP3 range in sequence identity to the DLP5 equivalents from 43.8% (gp28) to 93.2% (gp6), with all but gp28 having sequence identity greater than 70%. The gp28 proteins of DLP3 and DLP5 are related to the *Xylella* phage Sano tail fiber protein. The annotation of tail fiber for the Sano protein was based solely on synteny to the *Burkholderia* phage BcepNazgul255. There are no shared conserved domains identified between these two proteins, and the Bcep Nazgul protein is 1,270 amino acids long whereas the DLP3 and DLP5 gp28 proteins and the Sano tail fiber protein are only approximately 340 amino acids long; thus, we consider the assignment of tail fiber function to the Sano phage protein to be incorrect.

The gp28 proteins of DLP3 and DLP5 may play a role in host range due to the variability observed between these proteins. The DLP5 gp28 protein shares 62.5% identity with the Sano tail fiber, while DLP3 only has 39.6% identity to the Sano protein and 41.8% identity to the DLP5 protein. The first 242 N-terminal amino acids of the DLP3 and DLP5 gp28 consensus show a high degree of variability at 24.5% pairwise identity. The remaining 104 amino acids from 243 to the C-terminal have high pairwise identity at 88.5%. Variability in the N-terminal region was also observed with a MUSCLE alignment using all three proteins from DLP3, DLP5 and Sano (Supplementary Figure S1). The first 258 N-terminal amino acids of the protein consensus share only 35.4% pairwise identity, which increases to 77.8% over the remaining 104 amino acids. The alignment shows at least nine insertion/deletion events have occurred within the DLP3 gene resulting in gaps and insertions in the translated DLP3 protein compared to the Sano and DLP5 proteins. Further investigation into gp28 of DLP3 and DLP5 as a host recognition protein is currently underway to help elucidate whether this protein plays a role in the host range of the *Delepquintavirus* phages.

### Receptor Identification

**FIGURE 4:**
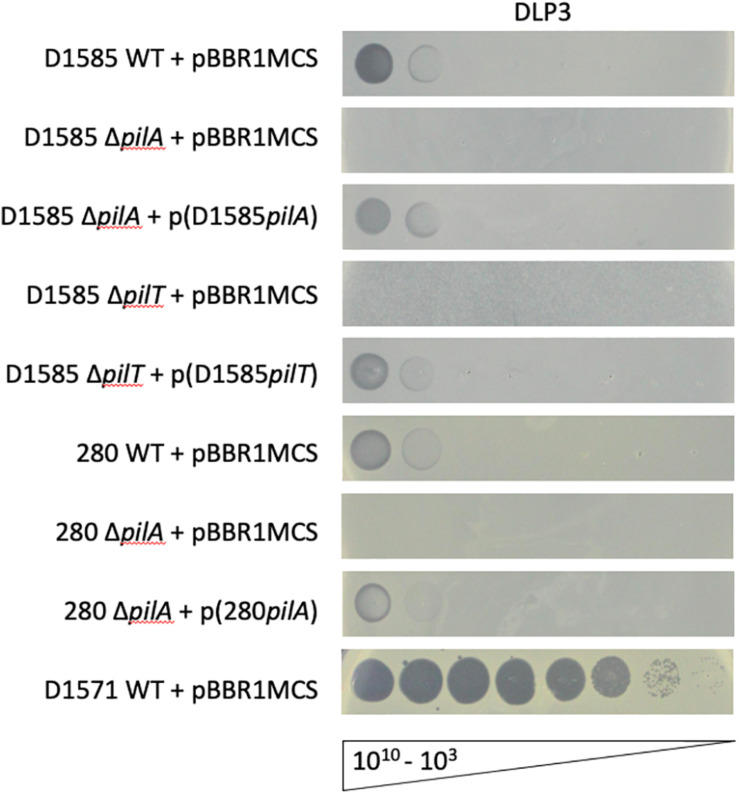
DLP3 requires a functional type IV pilus to infect host strains D1585 and 280. Phage DLP3 is capable of infecting wildtype *S. maltophilia* strains D1585 and 280, infecting both at 10^9^ PFU/mL, whereas phage infection is blocked in the Δ*pilA* and Δ*pilT* mutants. Complementation with the endogenous genes restores phage infection to wildtype levels. DLP3 plaquing ability on strain D1571 is shown for titer calculation, however, no mutants were constructed in this background. Images are representative of three biological replicates, each with three technical replicates.

Three *Siphoviridae* phages, DLP1, DLP2 and DLP4, previously isolated on *S. maltophilia* strain D1585, were found to bind the type IV pilus as their cell surface receptor across their host ranges (McCutcheon et al., 2018; Peters et al., 2019). Despite isolating DLP3 on strain D1571, we assessed DLP3 plaquing ability on the previously constructed D1585 and 280 Δ*pilA* mutants lacking the major pilin subunit. Similar to the three previously characterized phages, *S. maltophilia* strains lacking type IV pili are also resistant to infection by DLP3, shown by an absence of cell lysis at high titer (Figure 4). Complementation with the endogenous genes restores phage infection to wildtype levels. Unfortunately, the *pilA* gene is undetectable in the incompletely assembled D1571 genome, which prevented construction of pili mutants in this background. In contrast to host range data collected when DLP3 was initially isolated from the environment (Table 1), DLP3 efficiency of plating on D1585 and 280 is much lower than initially documented, showing phage activity at 10^9^ PFU/mL compared to plaque formation at 10^5^ PFU/mL (Figure 4). We attribute this reduced plaquing ability on some hosts to the repeated propagation of DLP3 on strain D1571. It is hypothesized that propagating DLP3 on a single host has selected for phages optimized to that host over time, resulting in the differences observed.

An additional mutant was constructed in strain D1585, Δ*pilT*, lacking the retraction ATPase required for depolymerization of the pilus (Burrows, 2005). This mutation results in hyperpiliated, non-motile cells having numerous non-functional pili projecting from the cell surface. Similar to the Δ*pilA* mutants, D1585 Δ*pilT* is resistant to DLP3 phage infection. This strain grows poorly in liquid, as observed in the speckled overlay lawn, however, this phenotype, as well as susceptibility to phage, is restored by complementation with the *pilT* gene (Figure 4). These results indicate that DLP3 uses the type IV pilus as a cell surface receptor and requires a functional pilus capable of retraction to reach the cell surface for successful host infection. This is the fourth documented *S. maltophilia* phage to use the type IV pilus as its receptor, however, we have yet to identify the receptor for the highly similar phage DLP5. Minor differences in the genomes of these two phages likely explains the significant differences observed in phage host range due to the binding of different receptors.

### Analysis of DLP3 Structural Proteins

To further investigate DLP3 morphology, phage structural proteins were analyzed by HPLC-MS and screened against DLP3 proteins and the *Stenotrophomonas* database in UniProt. The SEQUEST results from searching all DLP3 proteins identified 21 (Table 4), though only 11 proteins were classified as virion morphogenesis using BLASTp and CD-Search data (Supplementary Figure S2). The most abundant protein isolated was the major capsid protein (gp6), which is the main structural component of a bacteriophage capsid (Hendrix and Johnson, 2012). The second most abundant protein found was the portal protein (gp1), which forms the entry site for phage DNA to be packaged into the capsid by the large terminase. The portal protein also functions like a DNA sensor, measuring the amount of DNA packaged into the capsid and signaling the large terminase to end genome-packaging once full (Lokareddy et al., 2017).

**TABLE 4:** Mass spectrometry protein results with the DLP3 protein database.

Gp	Function	Score	Coverage (%)	Unique Peptides	Peptides	PSMs	AAs	MW [kDa]	calc. pI
6	Major capsid protein	1635.14	91.94	27	27	2709	310	33.7	5.76
14	Hypothetical protein gp_015	189.02	56.57	12	12	282	251	27.4	4.92
1	Portal protein	95.51	51.24	35	36	339	566	62.7	5.41
18	Hypothetical protein gp_019	54.48	19.54	10	10	123	527	58.2	6.27
17	Tape measure protein	45.75	21.85	28	28	78	1254	131.9	6.39
27	Tail assembly protein	33.34	28.48	7	7	46	323	35.4	7.05
19	DUF4302 family protein	30.84	27.47	7	7	69	324	35.8	6.61
24	Tail protein	23.39	26.51	18	18	106	826	90.1	5.08
97	WcaG	22.78	30.99	10	10	52	313	34.8	5.99
21	Minor tail protein	11.39	28.68	9	9	19	272	30.5	7.33
2	ParB-like nuclease domain protein	11.28	20.45	8	8	19	445	49.3	6.96
9	Hypothetical protein gp_010	8.01	31.03	6	6	11	203	22.5	5.94
28	Tail protein	7.74	24.71	10	10	24	340	37.2	8.73
20	Hypothetical protein gp_021	7.56	14.85	9	9	30	559	63.5	5.25
61	Hypothetical protein gp_066	6.45	27.45	7	7	31	204	22.3	9.00
25	Tail assembly protein	4.15	23.80	6	6	15	332	35.5	5.15
12	Hypothetical protein gp_013	2.37	78.35	4	4	7	97	9.6	9.47
3	Serine protease	2.24	9.95	2	2	3	201	22.7	4.67
57	RecA	2.03	15.56	5	5	7	405	43.5	6.46
5	Hypothetical protein gp_006	0.00	6.67	2	2	4	405	43.4	5.39
133	ATPase family protein	0.00	10.67	4	4	9	403	44.6	5.80

*Results are ordered by score.*

Gene products gp14 and gp18 were also identified. Both gene products are hypothetical proteins without conserved domains. Also, they do not have significant results using HHpred; therefore, their structural function is unknown (Table 4). Phyre2 analysis of these proteins only suggested a putative function for gp14, which showed structural identity to the major tropism determinant P1 (MTD-P1) of *Bordetella* phage BPP-1 (38.7% confidence, 25% identity). The MTD-P1 protein in BPP-1 is responsible for phage receptor binding (Miller et al., 2008) and uses specific variable residues for target recognition similar to an antibody (Miller, 2006). Another abundant protein identified is gp24, which is predicted to be a structural component of the DLP3 tail with a phage-tail_3 family (PF13550) conserved domain. The tape measure protein (gp17), which is responsible for setting the tail length (Belcaid et al., 2011), is the largest protein identified and the sixth most abundant, though the ∼130 kDa band was faint after fully destaining (Supplementary Figure S2). The remaining DLP3 proteins identified and ordered by relative abundance are gene products gp19, gp97, gp27, gp61, gp20, gp28, gp2, gp21, gp25, gp9, gp133, gp12, gp57, gp5, and gp3. Some of the proteins identified are surprising, such as gp2 (Par-B like nuclease domain protein) and gp5 (RecA), but based on the results obtained by screening the *Stenotrophomonas* protein database, it is evident that there were proteins present from the bacterial cell; thus, there may also be DLP3 proteins present that are not structural in function (Table 4 and Supplementary Table S2).

### Lysogenic Conversion of D1571 by Temperate Phage DLP3

Stable DLP3 lysogens of *S. maltophilia* strain D1571 have been isolated and due to the presence of ParB domains in two of the DLP3 proteins, DLP3 may lysogenize its host as a phagemid. The closely related phage DLP5 was also found to encode two proteins with ParB domains and was successfully isolated as a phagemid from the lysogenized strain D1614 (Peters and Dennis, 2018). Blastn and PHAST analysis (Altschul et al., 1997; Zhou et al., 2011) of GenBank-deposited *Stenotrophomonas* genomes indicates that phage DLP3 (or phages closely related to DLP3) has also lysogenized *S. maltophilia* strains ABB550, FDAARGOS_325 and ICU331. Phage DLP5 has been previously shown to cause lysogenic conversion of *S. maltophilia* strain D1571 (Peters and Dennis, 2018). Therefore, we sought to further investigate the characteristics of the D1571:DLP3 lysogen relative to wildtype *S. maltophilia* strain D1571.

### Identification of Erythromycin Resistance Factor Erm(45)

Annotation of the DLP3 genome revealed the presence of a methyltransferase domain in the gene product encoded by DLP3_099. A specific domain identified is the AdoMet_MTases superfamily comprised of class I S-adenosylmethionine-dependent methyltransferases. The class I family is the largest and most diverse, with members targeting a range of substrate specificities such as small molecules, lipids, nucleic acids, and different target atoms for methylation (Struck et al., 2012). Members of this family are known to maintain structural similarity even when the amino acid sequence diverges to as little as 10% (Schubert et al., 2003). Further investigation into gp69 with HHblits revealed high sequence identity to Erm(45) of *Staphylococcus fleurettii* (HHblits: 100% probability, 2.4e-104 *E*-value). Researchers identified Erm(45) as the enzyme responsible for increased erythromycin resistance in some strains of *S. fleuretti* (Wipf et al., 2015). The erm gene (erythromycin ribosome methylase) encodes a methylase enzyme that provides macrolide resistance by methylating the erythromycin target-site on the ribosome (Prunier et al., 2002). Together, these findings suggested that the DLP3 encoded methyltransferase is an erythromycin resistance protein similar to Erm(45) and could cause an increase in erythromycin resistance of the host cell during lysogeny.

**FIGURE 5:**
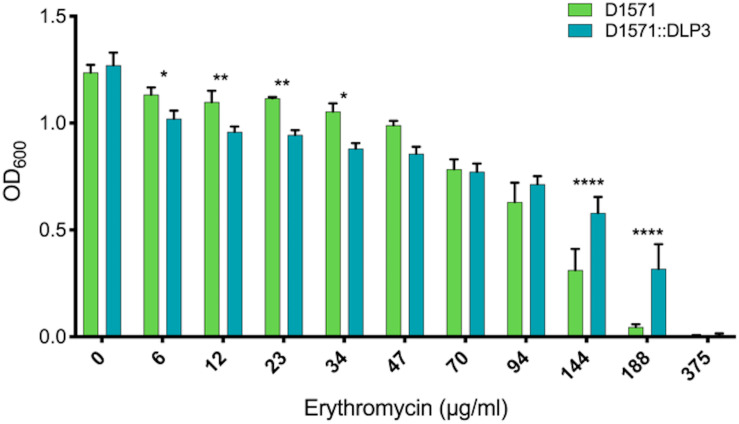
Erythromycin resistance of D1571:DLP3 lysogen increases compared to wild type control D1571. Minimum inhibitory concentration assay was completed in biological and mechanical triplicates. Two-way ANOVA with Sidak’s multiple comparisons test was performed on the MIC data. Statistical significance is represented as: ****P < 0.0001; **P < 0.01; and *P < 0.05.

To test the functionality of the DLP3 Erm protein, minimum inhibitory concentration (MIC) assays of the wild type D1571 and lysogen D1571:DLP3 were completed. The results show a statistically significant difference in erythromycin resistance with DLP3 lysogeny over a wide range of concentrations tested (Figure 5). There are differences noted between the wild type and lysogenized strains at lower concentrations of erythromycin, with wild type having a statistically significant increase in optical density at lower concentrations (6 and 34 μg/ml; *P* < 0.05, 12 and 23 μg/ml; *P* < 0.01). The cause of the decreased resistance in the lysogen noted at lower concentrations of erythromycin is currently unknown. This trend is reversed at higher concentrations of erythromycin, with the lysogen having significantly higher OD_600_ readings at 144 and 188 μg/ml erythromycin concentrations compared to wild type control (*P* < 0.0001). The increased resistance noted at higher erythromycin concentrations indicates lysogenic conversion of D1571 by lysogenized DLP3 and confirms the functionality of the predicted Erm protein. Fortuitously, *S. maltophilia* strains are already highly resistant toward macrolides, and macrolides such as erythromycin are not considered to be a frontline treatment option.

### Differing Growth Characteristics of DLP3 Lysogen *in vitro*

**FIGURE 6:**
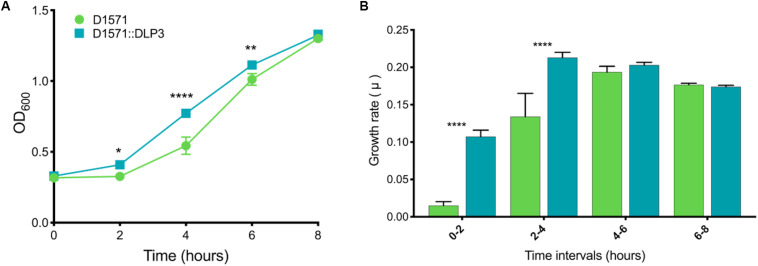
*In vitro* growth analysis of D1571 wild type and D1571:DLP3 lysogen. **(A)** Growth curve of wildtype D1571 compared to D1571:DLP3 strains grown in LB broth over 8 h. Results from biological and mechanical triplicate experiments were averaged and the mean plotted with the standard deviation represented as error bars. **(B)** Growth curve OD_600_ data was converted to the growth rate for wildtype and DLP3 lysogen with the mean plotted and error bars representing standard deviation. Two-way repeated measures ANOVA with multiple comparisons indicates statistical significance (*P < 0.05; **P < 0.01; ****P < 0.0001).

While working with the D1571:DLP3 lysogen and wild type D1571 for the erythromycin resistance assay, it was evident that the lysogen exhibited a faster growth rate as compared to the wild type strain. This difference in growth rate was also observed when growing single colony isolates of the lysogen and wild type on an LB agar plate, with the lysogen having larger single colonies after a 16 h incubation at 30°C as compared to the wild type D1571 (data not shown). To investigate this phenotypic difference further, a growth curve was conducted in mechanical and biological triplicates for the lysogen and wild type over 8 h. The plotted growth curve clearly shows the lysogenized strain exhibiting a faster growth rate than the wild type strain, with significance found following a two-way repeated measures ANOVA with multiple comparisons at 2 h, *P* < 0.05; 4 h, *P* < 0.0001; and 6 h, *P* < 0.01 (Figure 6A). This observation was confirmed by converting the OD_600_ data into growth rate (μ) and plotting the resulting data (Figure 6B). A two-way repeated measures ANOVA with multiple comparisons revealed significant (*P* < 0.0001) differences between 0- to 2-h and 2- to 4-h time intervals for the lysogen and wild type D1571. The increased growth rate is only observed during the lag and early exponential phase of growth and disappears in the 4- to 6-h and 6- to 8-h growth rate intervals. The cause of the growth rate differences observed with DLP3 lysogenization is currently unknown, though DLP3 does encode many hypothetical and moron genes which may be expressed to produce this phenotype.

### Virulence of D1571:DLP3 and DLP3 Rescue in *G. mellonella*

**FIGURE 7:**
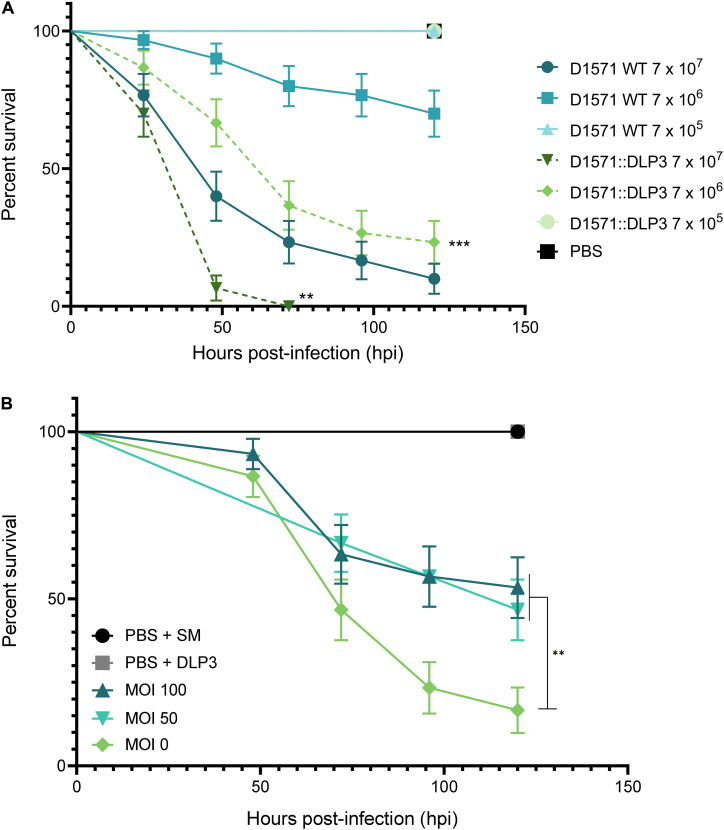
Effect of DLP3 on the virulence of D1571 in *G. mellonella.* **(A)** Survival of *G. mellonella* larvae over 120 h following infection with *S. maltophilia* D1571 wildtype or D1571:DLP3 lysogen at varying CFU. Larvae infected with D1571:DLP3 showed significantly lower survival at 10^7^ (**P < 0.01) and 10^6^ (***P < 0.001) CFU than larvae injected with the same inoculum of D1571 (log-rank test). **(B)** Survival of *G. mellonella* larvae injected with 8 × 10^8^CFU of D1571 over 120 h treated with DLP3 at an MOI of 100 (8.9 × 10^8^ PFU), 50 (4.5 × 10^8^ PFU) or 0 at 1.5 h post-infection. For controls, sterile PBS and SM were used in place of bacteria and phage injections, respectively. A significant difference in survival was observed between untreated larvae (MOI 0) and either phage treatment (**P < 0.01; log-rank test). Ten larvae were injected per group and results were obtained from three separate trials and plotted using the Kaplan–Meier method with standard error bars.

Based on the differing growth characteristics of D1571:DLP3 lysogen and the wildtype strain, we examined if the increased growth rate of the lysogen observed *in vitro* affected the virulence of strain D1571 *in vivo* using the *G. mellonella* larvae infection model. *G. mellonella* have been used as a non-mammalian eukaryotic model for assessing the virulence of many bacterial pathogens, including *S. maltophilia* (Betts et al., 2014; Tsai et al., 2016; Melloul et al., 2018), as well as a model to study the efficacy of novel antimicrobial compounds and phages (Seed and Dennis, 2009; Kamal and Dennis, 2015; Cutuli et al., 2019). Injection of *G. mellonella* larvae with *S. maltophilia* D1571 results in dose-dependent killing, with the lethal dose for this strain greater than 10^7^ CFU per larva (Figure 7A). Coinciding with the faster growth rate observed *in vitro*, the D1571:DLP3 lysogen was more virulent than the wildtype D1571 strain, resulting in significantly lower survival for *G. mellonella* larvae injected with 10^7^ (*P* < 0.01) or 10^6^ (*P* < 0.001) CFU over 120 h (Figure 7A). This increased virulence may be due to the faster growth rate of D1571:DLP3, however, CFU were not recovered from the larvae following infection.

Despite the DLP3 lysogen showing increased virulence *in vivo*, we sought to determine if DLP3 could rescue *G. mellonella* from wildtype D1571 infection. An inoculum of approximately 10^6^ CFU per larva was chosen to allow for phage rescue at a multiplicity of infection of at least 100, as DLP3 does not propagate higher than 10^10^ PFU/mL. Larvae were injected with DLP3 lysate at 1.5 h post-infection with D1571 and survival monitored over 120 h. Compared to treatment with SM buffer, significantly more larvae survived when given phage at a MOI of 50 or 100 (Figure 7B, *P* < 0.01), with an average survival at 120 h of 53% or 47% for worms treated at an MOI of 100 or 50, respectively, compared to 17% survival of untreated larvae. Attempts to concentrate DLP3 to a titer greater than 10^12^ PFU/mL without causing melanization of larvae following phage injection were unsuccessful, however, we expect that treatment at a higher MOI would increase the survival of infected larvae. Increased survival may also occur with repeated phage injections over the course of the experiment, however, this was not tested. Overall, this preliminary investigation using *G. mellonella* indicates the potential of DLP3 as a therapeutic for the treatment of *S. maltophilia* infections.

## Conclusion

The novel temperate phage DLP3 isolated from a soil sample enabled the identification of a second member of the newly established *Delepquintavirus* genus. DLP3 has a genome size of 96,852 bp and a GC content of 58.4%, which is significantly lower than the host strain D1571 which has a GC content of 66.9%. DLP3 encodes two proteins with ParB conserved domains enabling the stable lysogenization of the host strain D1571. Lysogenization by DLP3 leads to a growth rate increase during the lag and early exponential phase of growth for the host when compared to the wild type strain. DLP3 also encodes a functional Erm protein, allowing for the lysogenic conversion of the host D1571 strain which is observed though increased resistance to erythromycin at 144 and 188 μg/ml concentrations. Despite the temperate lifestyle of this phage, DLP3 is capable of lytic activity *in vivo*.

Like DLP3, many of the *S. maltophilia* temperate phages characterized to date feature moron genes that encode virulence factors or antibiotic resistance proteins that could cause lysogenic conversion of their host (Hagemann et al., 2006; Garcia et al., 2008; Peters and Dennis, 2018; Peters et al., 2019). Genetically modifying these phages by removing adverse genes, such as those encoding virulence factors or lysogeny proteins, will enable the creation of safe and highly targeted therapeutic agents (Lynch et al., 2010; Dedrick et al., 2019). Modified phages have been used in a case study against *Mycobacterium abscessus* (Dedrick et al., 2019) and a clinical trial (NCT04191148) sponsored by Locus Bioscience treating urinary tract infections with their modified crPhage cocktail. The genetically modified phage therapy examples set the precedence for other phages, such as DLP3, to be genetically modified and included in therapeutic phage cocktails. To this end, further study of DLP3 receptor binding proteins will possibly allow for the modification of other *S. maltophilia* phages showing therapeutic potential to target a wider host range.

## Data Availability Statement

The datasets generated for this study can be found in GenBank under accession number MT110073.

## Author Contributions

DP isolated phage DLP3, performed experiments to characterize the genome and viral particle, wrote the original manuscript draft and edited the manuscript. JM performed experiments to characterize the virus, wrote parts of the manuscript, and edited the manuscript. JD conceived of the research, directed the experiments, funded the research, and edited the manuscript. All authors contributed to the article and approved the submitted version.

## Conflict of Interest

The authors declare that the research was conducted in the absence of any commercial or financial relationships that could be construed as a potential conflict of interest.

## Supplementary Material

FIGURE S1 MUSCLE multiple sequence alignment of gp28 proteins from phages DLP3 and DLP5, and tail fiber protein of *Xylella* phage Sano. Note the increased amino acid conservation toward the C-termini of the proteins versus the N-termini as observed through protein alignment.

FIGURE S2 SDS-PAGE gel of DLP3 ghost particles (L) compared to a PageRuler Plus Pre-stained Protein Ladder (Thermo Scientific). Approximate masses are labeled on the right of the gel in kDa. Protein bands labeled using mass spectrometry results from SEQUEST scan of DLP3 and *Stenotrophomonas* protein databases. Stacking and resolving portions of the gel were 4 and 7.5%, respectively. The gel was stained with Coomassie R-250.

TABLE S1 Bacterial strains and plasmids used in this study.

TABLE S2 Mass spectrometry protein results using the Stenotrophomonas protein database in UniProt. The results are organized by score.
