# Supplementary figures and images for: Characterization of Novel Broad-Host-Range Bacteriophage DLP3 Specific to Stenotrophomonas maltophilia as a Potential Therapeutic Agent
Source: Front Microbiol. 2020 Jun 24;11:1358. doi: 10.3389/fmicb.2020.01358 (PMC7326821; doi:10.3389/fmicb.2020.01358)

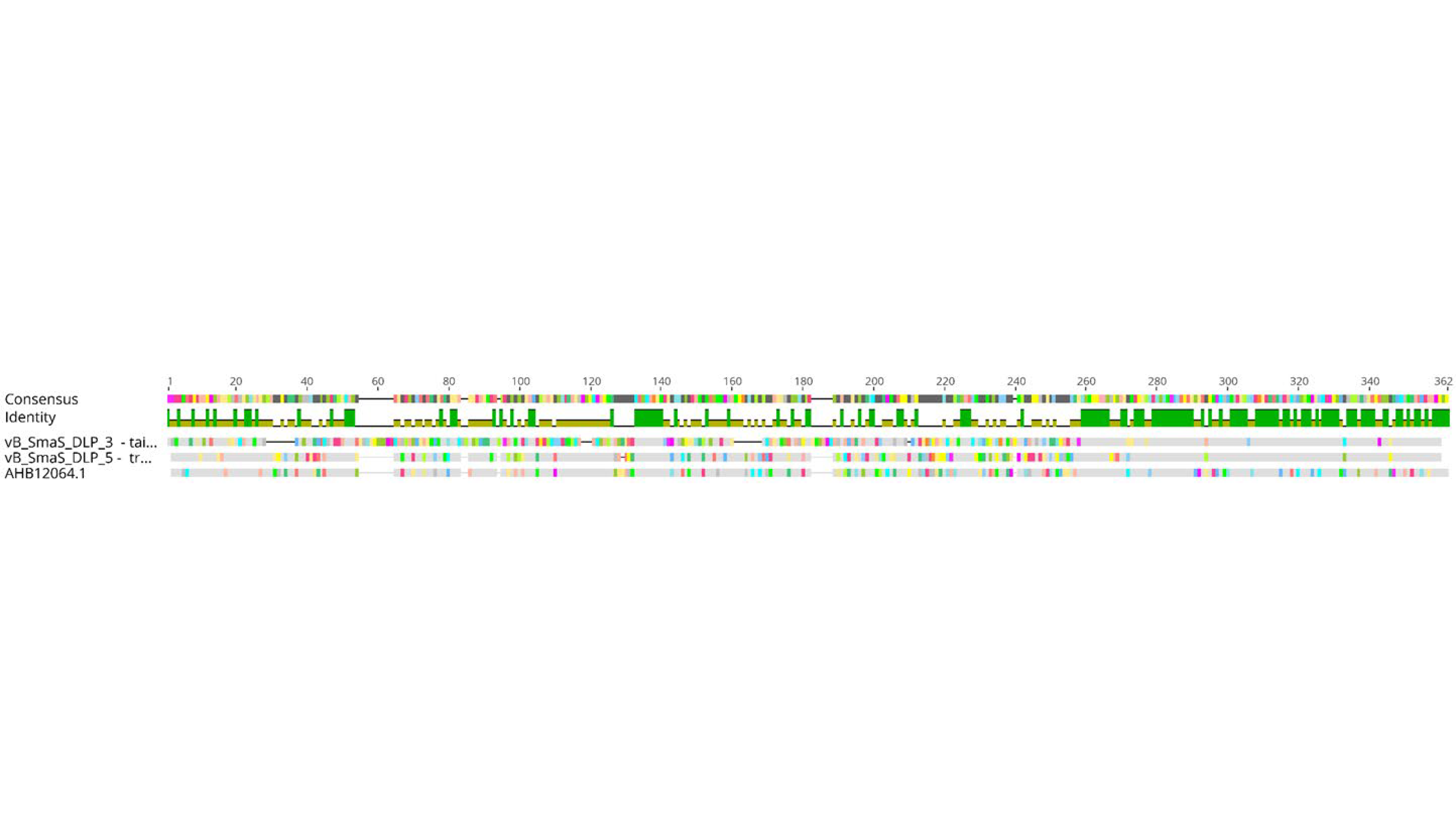

Supplement: FIGURE S1 — MUSCLE multiple sequence alignment of gp28 proteins from phages DLP3 and DLP5, and tail fiber protein of Xylella phage Sano. Note the increased amino acid conservation toward the C-termini of the proteins versus the N-termini as observed through protein alignment. [file Image_1.tif]

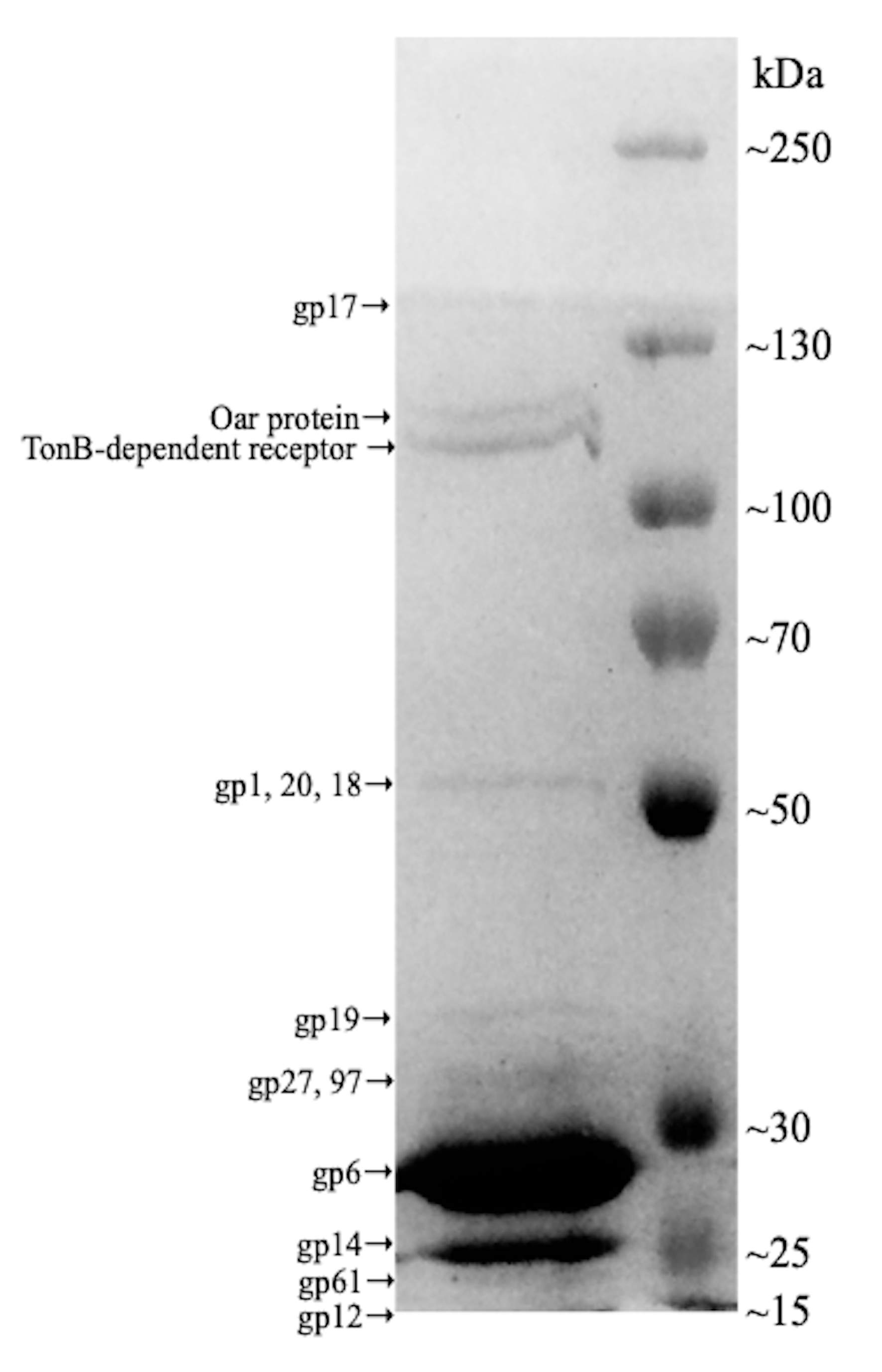

Supplement: FIGURE S2 — SDS-PAGE gel of DLP3 ghost particles (L) compared to a PageRuler Plus Pre-stained Protein Ladder (Thermo Scientific). Approximate masses are labeled on the right of the gel in kDa. Protein bands labeled using mass spectrometry results from SEQUEST scan of DLP3 and Stenotrophomonas protein databases. Stacking and resolving portions of the gel were 4 and 7.5%, respectively. The gel was stained with Coomassie R-250. [file Image_2.TIF]
