# Supplementary material for: Characterization of Novel Broad-Host-Range Bacteriophage DLP3 Specific to Stenotrophomonas maltophilia as a Potential Therapeutic Agent
Source: Front Microbiol. 2020 Jun 24;11:1358. doi: 10.3389/fmicb.2020.01358 (PMC7326821; doi:10.3389/fmicb.2020.01358)
Supplement: TABLE S1 — Bacterial strains and plasmids used in this study. [file Table_1.pdf]

Suppl. Table 1: Bacterial strains and plasmids used in this study.

**Bacterial Strain**	**Genotype or Description**	**Source**
D1585 Δ*pilA*	Clean deletion of *pilA* in D1585	McCutcheon et al., 2018
D1585 Δ*pilT*	Clean deletion of *pilT* in D1585	This study
280 Δ*pilA*	Clean deletion of *pilA* in 280	McCutcheon et al., 2018
E. coli S17-1	Conjugative donor strain	Simon et al., 1983
E. coli DH5α	Host for plasmid cloning	Hanahan et al., 1991
**Plasmids**		
pBBR1MCS	Broad-host range cloning vector, Cm^R	Kovach et al., 1994
pD1585pilA	pBBR1MCS carrying D1585 *pilA*, Cm^R	McCutcheon et al., 2018
pD1585pilT	pBBR1MCS carrying D1585 *pilT*, Cm^R	This study
p280pilA	pBBR1MCS carrying 280 *pilA*, Cm^R	McCutcheon et al., 2018
pEX18Tc	Tc^R, *oriT*, *sacB*, gene replacement vector	Hoang et al., 1998
pD1585ΔpilT	pEX18Tc, 2 kb ΔpilT D1585 region	This study
