# Supplementary material for: Characterization of Novel Broad-Host-Range Bacteriophage DLP3 Specific to Stenotrophomonas maltophilia as a Potential Therapeutic Agent
Source: Front Microbiol. 2020 Jun 24;11:1358. doi: 10.3389/fmicb.2020.01358 (PMC7326821; doi:10.3389/fmicb.2020.01358)
Supplement: TABLE S2 — Mass spectrometry protein results using the Stenotrophomonas protein database in UniProt. The results are organized by score. [file Table_2.pdf]

Suppl. Table 2: Mass spectrometry protein results using the *Stenotrophomonas* protein database in UniProt. The results are organized by score.

Hit	Gene	Score	Coverage (%)	Proteins	Unique Peptides	Peptides	PSMs	AAs	MW [kDa]	calc. pI
TonB-dependent receptor	B9Y71_14085	127.60	23.45	3	20	20	94	985	105.6	6.42
Oar protein	BWP19_02175	115.44	22.70	3	22	22	84	1075	118.0	6.48
TonB-dependent receptor	AR275_14455	72.57	22.09	1	18	18	64	987	106.9	5.36
Putative TonB dependent receptor	Smlt3478[a]	69.96	14.94	1	12	12	43	944	100.4	5.67
TonB-dependent receptor	BWP19_04650	49.49	11.48	2	10	10	26	1028	111.9	6.46
Porin	B9Y71_15810	48.38	29.56	3	11	11	25	389	42.9	6.64
Uncharacterized protein	AR275_04505	41.26	38.25	1	11	11	27	366	39.5	5.00
TonB-dependent receptor	BWP19_01190	39.51	18.17	4	15	15	31	952	103.0	5.83
Ligand-gated channel	YH67_15830	36.92	15.29	11	10	10	19	811	87.5	5.59
TonB-dependent receptor	YH67_02440	32.07	11.49	20	10	10	22	879	94.4	6.62
Probable cytosol aminopeptidase	pepA	30.69	12.40	11	6	6	17	492	51.2	5.52
Uncharacterized protein	B9Y76_18325	26.74	37.13	1	7	7	14	272	30.0	5.41
Dihydrolipoyl dehydrogenase	B9Y71_11945	23.96	14.85	4	7	7	17	478	50.4	6.70
Protein CyaE	B9Y71_15750	22.44	17.04	13	6	6	12	452	48.7	6.46
Membrane protein	ABW44_09025	19.18	14.89	1	5	5	11	450	47.2	6.55
50S ribosomal protein L2 (Fragment)	VM57_03345	16.67	23.74	5	4	4	10	198	21.3	9.99
DNA-directed RNA polymerase subunit	L681_20760[c]	16.51	8.99	28	7	7	10	790	88.9	8.97
Uncharacterized protein	B9Y57_19230	15.88	38.83	1	6	6	10	206	22.6	8.29
TonB-dependent receptor	VL23_08990	13.73	9.52	18	7	7	10	756	82.7	5.36

30S ribosomal protein S13	rpsM	13.36	31.36	3	3	3	6	118	13.4	11.46
TonB-dependent receptor	STRNTR1_0356	13.15	5.98	24	6	6	7	953	103.1	5.45
OmpW family protein	SmaCSM2_17180	12.97	20.57	21	3	3	12	209	22.1	8.76
TonB-dependent receptor	BWP19_04640	11.53	7.49	3	5	6	10	935	101.8	6.30
Esterase	AR275_13845	11.17	8.51	3	4	4	7	611	64.1	6.55
Cell surface protein	BWP19_07340	10.67	2.47	6	5	5	9	2393	223.5	4.59
Autotransporter-associated beta strand	A1OC_03319[b]	9.41	4.87	4	4	4	5	944	96.6	8.22
TonB-dependent receptor	AR275_02775	8.50	13.05	5	7	7	8	613	66.5	6.65
Porin	SmaCSM2_17905	8.17	11.08	4	4	4	4	379	41.9	7.08
Malic enzyme (Fragment)	AR275_14665	7.89	3.22	22	2	2	4	652	70.9	6.19
30S ribosomal protein S3	rpsC	7.46	23.24	5	4	4	5	241	27.2	10.15
TonB-dependent receptor	ABW44_04685	7.41	7.55	7	5	6	8	940	103.1	5.66
TonB-dependent receptor	B9Y56_09770	7.28	3.15	2	3	3	5	1047	114.4	5.57
Outer membrane receptor protein	BB780_13710	5.97	2.94	7	2	2	3	749	80.7	6.51
TonB-dependent receptor	BWP19_12960	5.50	2.96	19	3	3	5	912	100.4	5.60
Uncharacterized protein	CR919_19680	4.41	3.96	23	2	2	2	732	79.3	5.59
ATP synthase gamma chain	atpG	4.31	8.36	8	2	2	2	287	31.9	9.61
TldD protein (Fragment)	AR275_13300	4.18	4.52	9	2	2	2	442	47.4	8.07
Ribonucleoside-diphosphate reductase	RRM1[a]	4.07	5.68	14	3	3	3	775	86.7	6.57

Autotransporter outer membrane beta-barrel domain-containing protein	B9Y56_12910	3.83	2.65	1	2	2	2	791	82.4	6.06
Oar protein	BWP19_04665	3.79	2.68	2	2	2	2	1007	109.5	5.81
Uncharacterized protein	ARC23_12465	3.73	3.05	1	3	3	3	985	108.6	5.47
TonB-dependent receptor	AR275_05645	3.73	5.08	8	3	3	3	768	82.6	6.57
Flagellin	flic7[d]	3.67	6.67	8	2	2	3	390	40.0	5.40
TonB-dependent receptor (Fragment)	ARC78_03340	3.63	3.52	31	2	2	3	739	79.3	5.22
30S ribosomal protein S2	rpsB	3.61	10.49	9	3	3	4	267	29.4	8.51
TonB-dependent receptor	ABW44_01270	3.60	4.59	2	4	4	5	806	88.6	6.46
TonB-dependent receptor	YH67_13890	3.51	5.15	27	4	4	4	776	84.5	6.10
Porin	B9Y61_10620	3.44	4.00	22	2	2	2	475	50.9	6.09
Peptidase M20	YH67_02165	3.37	7.29	22	3	3	3	549	59.7	6.43
Uncharacterized protein	u35[d]	2.52	6.69	2	3	3	3	658	71.5	5.50
ATP synthase subunit alpha	atpA	1.89	3.88	1	2	2	2	515	55.3	5.62
Uncharacterized protein (Fragment)	BWP19_21350	1.80	9.52	9	2	2	2	210	23.0	4.65
Histidine ammonia-lyase	hutH	1.79	5.85	7	3	3	3	513	53.5	5.69
OmpA-related protein	SmaCSM2_14405	1.71	3.73	15	3	3	4	1018	110.4	6.37

[a]K279a, [b]Ab55555, [c]MF89, [d]RA8
